# Definition of metafounders based on population structure analysis

**DOI:** 10.1186/s12711-024-00913-7

**Published:** 2024-06-06

**Authors:** Christine Anglhuber, Christian Edel, Eduardo C. G. Pimentel, Reiner Emmerling, Kay-Uwe Götz, Georg Thaller

**Affiliations:** 1 Bavarian State Research Center for Agriculture, Institute for Animal Breeding, Prof. Duerrwaechter Platz 1, 85586 Grub, Germany; 2 Institute for Animal Breeding and Husbandry, Christian-Albrechts-Universität, Olshausenstraße 40, 24098 Kiel, Germany

## Abstract

**Background:**

Limitations of the concept of identity by descent in the presence of stratification within a breeding population may lead to an incomplete formulation of the conventional numerator relationship matrix ($\mathbf{A}$). Combining $\mathbf{A}$ with the genomic relationship matrix ($\mathbf{G}$) in a single-step approach for genetic evaluation may cause inconsistencies that can be a source of bias in the resulting predictions. The objective of this study was to identify stratification using genomic data and to transfer this information to matrix $\mathbf{A}$, to improve the compatibility of $\mathbf{A}$ and $\mathbf{G}$.

**Methods:**

Using software to detect population stratification (ADMIXTURE), we developed an iterative approach. First, we identified 2 to 40 strata ($k$) with ADMIXTURE, which we then introduced in a stepwise manner into matrix $\mathbf{A}$, to generate matrix ${\mathbf{A}}^{{\varvec{\Gamma}}}$ using the metafounder methodology. Improvements in consistency between matrix $\mathbf{G}$ and ${\mathbf{A}}^{{\varvec{\Gamma}}}$ were evaluated by regression analysis and through the comparison of the overall mean and mean diagonal values of both matrices. The approach was tested on genotype and pedigree information of European and North American Brown Swiss animals (85,249). Analyses with ADMIXTURE were initially performed on the full set of genotypes (S1). In addition, we used an alternative dataset where we avoided sampling of closely related animals (S2).

**Results:**

Results of the regression analyses of standard $\mathbf{A}$ on $\mathbf{G}$ were – 0.489, 0.780 and 0.647 for intercept, slope and fit of the regression. When analysing S1 data results of the regression for ${\mathbf{A}}^{{\varvec{\Gamma}}}$ on $\mathbf{G}$ corresponding values were – 0.028, 1.087 and 0.807 for $k$=7, while there was no clear optimum $k$. Analyses of S2 gave a clear optimal $k$=24, with − 0.020, 0.998 and 0.817 as results of the regression. For this $k$ differences in mean and mean diagonal values between both matrices were negligible.

**Conclusions:**

The derivation of hidden stratification information based on genotyped animals and its integration into $\mathbf{A}$ improved compatibility of the resulting ${\mathbf{A}}^{{\varvec{\Gamma}}}$ and $\mathbf{G}$ considerably compared to the initial situation. In dairy breeding populations with large half-sib families as sub-structures it is necessary to balance the data when applying population structure analysis to obtain meaningful results.

**Supplementary Information:**

The online version contains supplementary material available at 10.1186/s12711-024-00913-7.

## Background

In genomic predictions, especially when using the single-step approach for genomic predictions, it is important to adjust the standard numerator relationship matrix $\mathbf{A}$ and the genomic relationship matrix $\mathbf{G}$, so that both matrices refer to the same genetic base and can consequently be regarded as covariance matrices with the same genetic variance [1]. Differences in the underlying assumptions for the construction of these matrices lead to different properties of these matrices that might be a source of bias when they are combined in a single model. For example, identity by descent (IBD) is the underlying concept in the construction of the numerator relationship matrix $\mathbf{A}$, and inbreeding describes the decrease in heterozygosity initially presumed to be at maximum for a group of $n$ base animals (the base population comprises an infinite number of independent loci, each with 2 $n$ unique alleles [2]). This concept cannot be easily transferred to a matrix $\mathbf{G}$ that is calculated from $m$ biallelic SNP markers, irrespective of the way $\mathbf{G}$ is calculated. Moreover, in calculating $\mathbf{G}$ (e.g., VanRaden [3]), the distribution of genotypes and aspects like population subdivision or crossbreeding may additionally affect the structure of the matrix. This might lead to a situation, where the differences between both covariance matrices become critical, eventually leading to biased and/or unreliable estimates of genetic merit [4].

Several studies made suggestions how to best adjust $\mathbf{G}$ to fit $\mathbf{A}$ [5–8], accounting for genotyping strategies [9] or differences in assumptions about the implied base of the matrices [10]. Christensen [11] proposed to calculate $\mathbf{G}$ like in VanRaden [3], but assuming a homogeneous base population of maximum average heterozygosity under Hardy–Weinberg equilibrium (e.g., base allele frequencies of 0.5 for all biallelic loci involved in the calculation). This method of calculating $\mathbf{G}$ preserves important conceptual similarities with $\mathbf{A}$, e.g., a cumulative decrease in heterozygosity (all diagonal elements being > 1, given the population is homogeneous). However, in this situation $\mathbf{G}$ and $\mathbf{A}$ do not refer to the same base population and to the same genetic variance without further adjustments. To align both matrices Christensen [11] introduced the scaling parameter $\gamma$, which is a function of the (assumed) average heterozygosity of the pedigree base. This $\gamma$ is then used to scale $\mathbf{A}$ and its genetic variance so that it matches $\mathbf{G}$. This first attempt to harmonize a model based on pedigree information and a model based on genomic information was later on elaborated by Legarra et al. [12] to allow for several interbreeding conceptual base populations (metafounders). In its present state the metafounder concept is an elaborated methodological framework providing a general and consistent formulation of the genetic model underlying single-step genomic evaluations even in the presence of population subdivision, introgression, and crossbreeding. It has been tested so far in several applications for single breeds [13–15], multibreed [16–18] and crossbreed cattle [19–21] as well as in plants [22].

Admixed or structured populations show a separation into two or more subpopulations, that can be characterized based on their specific allele frequencies. Admixed populations may be well defined, e.g., in the case of model animals with well-documented pedigrees. On the other hand, many livestock populations are characterized by incomplete pedigrees and poorly documented introgression, making it difficult to properly define metafounders based on pedigree data alone. Several studies have used ADMIXTURE to investigate population stratification in human, animal (both wildlife and domestic) and plant populations [23–33]. It has successfully been used in domestic cattle populations to discover important steps in the history of domestication [24], to reveal the genetic background of northern red dairy cattle breeds [34] or to assess genetic diversity in conservation schemes [27, 32]. ADMIXTURE identifies anonymous stratification (i.e., stratifications not necessarily traceable to information available from pedigree data of an individual) from a set of genotyped animals by using a maximum-likelihood approach [35]. The European Brown Swiss breed is an example of a highly admixed population [36, 37]. First attempts to define metafounders based on the existing pedigree information did not lead to satisfactory results. Therefore, we are looking into approaches to derive population stratification directly from genotype data. The objective of this paper was to derive stratification information directly from genomic data using population structure analysis, and to transfer this information to the submatrix of genotyped animals (${\mathbf{A}}_{g}$) by using the theoretical framework of the metafounder concept, to improve the compatibility between ${\mathbf{A}}_{g}$ and $\mathbf{G}$. We illustrated our approach on genotype and pedigree data from the European Brown Swiss population and examined different methods to evaluate the compatibility of ${\mathbf{A}}_{g}$ and $\mathbf{G}$.

## Methods

### Basic concepts of the investigation

In our approach strata (metafounders) are abstract entities that may coincide with a real source of genetic variation (e.g., an ancestral breed). We derived the relevant information about the strata directly from genomic data using the ADMIXTURE software [35]. All other steps were performed using in-house scripts. To investigate the feasibility of the approach we developed an iterative workflow where we increased the number of strata ($k$) in a stepwise manner and introduced this information into matrix ${\mathbf{A}}_{g}$ using metafounder methodology to create matrix ${\mathbf{A}}^{{\varvec{\Gamma}}}$, a relationship matrix where founders can be related and inbred [12]. We then evaluated improvements in consistency between $\mathbf{G}$ and the resulting matrix ${\mathbf{A}}^{{\varvec{\Gamma}}}$ by visual inspection of graphs from principal component analyses, by regression analysis and through the comparison of overall means and mean diagonal values of both matrices.

In our approach we assigned genotyped animals directly to the strata via an estimated matrix $\mathbf{Q}$, which describes the gene-flow from these strata across the founders to the genotyped animals. This approach circumvents the need to assign a pedigree founder exclusively to one or two specific strata and each animal of the pedigree base may represent a complex mixture of genomic strata. This is different to the original metafounder approach, where pedigree founders are directly assigned to metafounders, based on registration or pedigree information [12].

### Detection of stratification information

ADMIXTURE had several advantages over other software available to identify population stratification. For instance, it did not need representatives of the ancestral origin in the data, and it was able to handle larger data sets [35]. ADMIXTURE estimates the $n$ x $k$ matrix $\mathbf{Q}$ describing the contributions of the *k* strata to each of the $n$ genotyped individuals. The number of strata must be provided by the user. ADMIXTURE additionally provides an $m$ x $k$ matrix ${\mathbf{P}}_{\text{A}}$ of estimated strata-specific allele frequencies, where $m$ stands for the number of provided marker loci.

ADMIXTURE offers two options, a supervised and an unsupervised mode. In the supervised mode, the investigator assigns individuals perceived as unadmixed, i.e., as representatives of a distinct origin, to a specific stratum. These individuals are used as reference in the analysis of genotypes. For this approach reliable information on the genetic background of assigned animals is crucial and each stratum must be represented by some animals. In the unsupervised mode, only the genotype data and the chosen number of strata ($$k)$$ is supplied to ADMIXTURE for detection of stratification. ADMIXTURE does not provide an estimate of the optimum number of genomic strata characterizing the sample. However, by using the ‘cv’ option, ADMIXTURE performs a tenfold cross-validation and the results of this cross-validation can be used as a criterion to choose an optimum value for $$k$$ [35]. This approach has some limitations. It has frequently been reported that ADMIXTURE is sensitive to familial structures in the material. Especially if the data comprises large half-sib groups no clear optimum value for $$k$$ from cross-validation can be discerned [24–26], which makes application in cattle populations difficult. Since our focus was to detect sources of stratification beyond the information already reflected by the standard relationship matrix $$\mathbf{A}$$, most of the familial information recovered by ADMIXTURE may be redundant, because pedigrees of the animals are usually available. However, there is no way to know beforehand which newly added stratum will provide information beyond the information already included in the pedigree, because ADMIXTURE provides no interpretation of the identified strata. To investigate the effect of close relationships in the data, we followed two different strategies in a strictly unsupervised manner. In strategy 1 we provided all genotyped animals to ADMIXTURE. In strategy 2 we provided ADMIXTURE with a dataset that minimized the degree of relationships of genotyped animals by sampling only one member of each half-sib family. For details of the selection process please refer to the data section. However, if ADMIXTURE is provided with a smaller sample, the estimated matrix $$\mathbf{Q}$$ no longer contains rows for all genotyped animals. Chiang et al. [38] developed a simple approach to derive admixture proportions based on allele frequencies and genotype data. To derive rows of $$\mathbf{Q}$$ for the remaining animals, we regressed one half of the genotype of each animal on the matrix $${\mathbf{P}}_{\text{A}}$$ [38, 39]:

1 $${\mathbf{q}}_{{\varvec{i}}}^{{\prime}}={(\mathbf{P}}_{\text{A}}^{{\prime}}{\mathbf{P}}_{\text{A}}{)}^{-1\boldsymbol{ }}{\mathbf{P}}_{\text{A}}^{{\prime}}\boldsymbol{ }{(0.5\mathbf{z}}_{{\varvec{i}}}^{{\prime}}),$$

where ${\mathbf{q}}_{{\varvec{i}}}^{{\prime}}$ is the vector of estimated strata $k$ contributions to animal $i$ and ${\mathbf{z}}_{{\varvec{i}}}^{{\prime}}$ is a vector of length $m$ of genotypes coded as 0,1,2 (allele counts for the reference allele) for animal $i$. In cases where the sum of estimated strata contributions to an animal exceeded 1 it was rescaled to 1 [40]. Matrix ${\mathbf{P}}_{\text{A}}$ can be augmented by a column to estimate a global intercept. A non-zero intercept-estimate can then be interpreted as twice the contribution of a stratum not considered so far [38]. A non-zero intercept for many animals in the analysis indicates that *k* is not enough to detect all relevant strata.

### Matrix $\mathbf{G}$

In this investigation we constructed the genomic relationship matrix $\mathbf{G}$ as the cross-product of a matrix of recoded numeric genotype counts $\mathbf{M}$ (e.g., VanRaden’s approach 1 [3]). In this approach, the scaled genotype count of a homozygote for the reference allele at an arbitrary locus is $2-1-2\left({p}_{B}-0.5\right)=2-2{p}_{B}=2{q}_{B}$, where ${p}_{B}$ is the frequency of the reference allele at that particular locus in the base population and ${q}_{B}=1 -{p}_{B}$. The resulting matrix is finally divided by a scaling parameter $c = \sum_{j=1}^{m}2{p}_{{B}_{j}}{q}_{{B}_{j}}$, which is the sum of expected genotype-frequencies of heterozygotes calculated from base population allele frequencies across all $m$ markers under the assumption of Hardy–Weinberg equilibrium (HWE). Therefore, recoded genotype counts as well as the final scaling parameter are functions of base allele frequencies that are assumed to be known. To simplify formulae in what follows we used ${H}_{B}= \overline{2{p }_{B}{q}_{B}}$ for the *average expected heterozygosity* over all $m$ markers in the base population, hence $c = m*{H}_{B}$.

Different methods to calculate the genomic relationship-matrix $\mathbf{G}$ have been described in literature [3, 41, 42]. No matter how **G** is calculated, it should preserve fundamental conceptual properties, e.g., it should be a valid coefficient matrix describing the covariance of Mendelian sampling terms. For the following considerations, we particularly require that a diagonal element of $\mathbf{G}$ must be proportional to $1+{F}_{T,B}$, where ${F}_{T,B}$ is the inbreeding coefficient of an animal $T$ relative to base population $B$. More formally, the diagonal element corresponding to animal $T$ should fulfill

2 $${\mathbf{G}}_{{\varvec{T}},{\varvec{T}}} = 1+{F}_{T,B }=1 + \left(1-{P}_{T,B}\right)= 1+\left(1-\frac{{H}_{T}}{{H}_{B}}\right)= 2 - \frac{{H}_{T}}{{H}_{B}},$$

where ${H}_{T}$ is the *average observed heterozygosity of animal T* (number of all heterozygous loci relative to all loci) and ${P}_{T,B}={H}_{T}/{H}_{B}$ is commonly referred to as the *panmictic index* of animal $T$ relative to the base $B$ [2], thus relating the individual heterozygosity of animal $T$ to the overall expected heterozygosity of the base population.

Assuming a fixed base population frequency of 0.5 for all loci in VanRaden’s approach 1 [3] when constructing $\mathbf{G}$ is a convenient way to guarantee that the above expectation (2) holds [3, 6, 11, 12]. This can be shown by equating (2) to its expectation under the assumption of a fixed base allele frequency of 0.5. Assuming a fixed base population allele frequency of 0.5 for all loci, results in the diagonal element of animal $T$ being equal to the number of homozygote loci it carries divided by $c$. This can equivalently be expressed as $m\left(1-{H}_{T}\right)/c$ and hence:

$${\mathbf{G}}_{{\varvec{T}},{\varvec{T}}}= 1+{F}_{T,B}$$

$$\frac{m\left(1-{H}_{T}\right)}{m*{H}_{B}}= 2 - \frac{{H}_{T}}{{H}_{B}}$$

$$\frac{\left(1-{H}_{T}\right)}{{H}_{B}}- 2+ \frac{{H}_{T}}{{H}_{B}}=0$$

$$2*{H}_{B} =1$$

$${H}_{B} =0.5$$

Therefore, the resulting diagonals fulfill (2) if ${H}_{B}=0.5$. If not otherwise indicated, in what follows, genomic relationship matrices are assumed to be calculated assuming ${p}_{B}=0.5$ for all markers involved in the calculation.

### Rescaling

If an individual inbreeding coefficient was calculated by relating an individual’s heterozygosity to the overall expected heterozygosity of a certain base population, it is possible to express it referring to another base population with different heterozygosity. In this case standard formulations based on panmictic indices can be used to transform individual inbreeding coefficients calculated with reference to a particular base heterozygosity (${H}_{B}$) to any other base population heterozygosity (${H}_{X}$) by [2]

$${F}_{T,X}= 1 - {P}_{T,B}{P}_{B,X} = 1 - \frac{{H}_{T}}{{H}_{B}} \frac{{H}_{B}}{{H}_{X}},$$

This approach can be extended to rescale complete relationship matrices, e.g., to transform a relationship matrix ${\mathbf{A}}_{B}$ relating to a defined average base-group heterozygosity $B$ to another base-group with a different average heterozygosity $X$ using

$${\mathbf{A}}_{X}= {P}_{B,X} ({\mathbf{A}}_{B} - 2) + 2,$$

In cases where $X$ refers to a hypothetical base population of maximum heterozygosity (${H}_{X}=0.5$), which implies a base allele frequency of 0.5 for all loci, this expression simplifies to

$${\mathbf{A}}_{X}= 2{H}_{B}({\mathbf{A}}_{B} - 2) + 2,$$

An equivalent formulation was derived by Christensen [11] and has been further elaborated by Legarra et al. [12] within the general framework of the metafounder concept. Both publications used the rescaling term $$\gamma$$, which can be interpreted as a single metafounder representing a base with inbreeding of $${\raise0.7ex\hbox{$\gamma $} \!\mathord{\left/ {\vphantom {\gamma 2}}\right.\kern-0pt} \!\lower0.7ex\hbox{$2$}}$$ [11] relative to a hypothetical base

$$\frac{\gamma }{2}={F}_{B,X}=1 - {P}_{B,X}$$

$$\gamma =2 \left(1 - {P}_{B,X}\right)=2 \left(1 - \frac{{H}_{B}}{{H}_{X}}\right)$$

If rescaling is to a base population of maximum heterozygosity (${H}_{X}=0.5$), this simplifies to $\gamma =2 \left( 1 - 2{H}_{B}\right)=4 (0.5-{H}_{B})$. Note, that when relationship matrices are used as coefficient matrices of an additive-genetic variance parameter, for each rescaling step the associated parameter estimate has to be rescaled accordingly [12].

### Strata allele frequencies and the calculation of ${\varvec{\Gamma}}$

Several methods have been proposed in literature to estimate the matrix of ‘self-relationships’ of Metafounders ${\varvec{\Gamma}}$ either directly or via the estimation of strata-specific allele frequencies [43]. Although ADMIXTURE provides estimates for strata allele frequencies, these are estimated from correlated observations that are not corrected for trivial sources of stratification like family structures that are already represented by the known pedigree. Adding this redundant stratification information to $\mathbf{A}$ would result in double counting of information. To compensate for this effect, we estimated allele frequencies based on both, the estimated matrix $\mathbf{Q}$ provided by ADMIXTURE, and the known pedigree represented by the numerator relationship matrix of genotyped animals ${\mathbf{A}}_{g}$, with the method presented by Gengler et al. [44] and further elaborated by Aldridge et al. [45] and Plieschke et al. [36].

3 $${\mathbf{p}}_{j}={0.5*[(\mathbf{Q}^{{\prime}}{\mathbf{A}}_{g}^{-1}\mathbf{Q})}^{-1}\mathbf{Q}^{{\prime}}{\mathbf{A}}_{g}^{-1}{\mathbf{z}}_{j}],$$

where ${\mathbf{p}}_{j}$ is a vector of estimated allele frequencies for marker $j$ and ${\mathbf{A}}_{g}^{-1}$ is the inverse of the submatrix of the genotyped animals and ${\mathbf{z}}_{j}$ is a vector of genotypes coded as 0,1,2 for all animals at marker $j$. Generalized least squares (GLS) approaches to estimate allele frequencies like (3) have been shown to provide unbiased estimates under quite general conditions [43]. Finally, ${\varvec{\Gamma}}$ was constructed as described in [43] with allele frequencies estimated with Eq. (3) and relating to a base population of maximum heterozygosity under HWE [43]:

4 $${\varvec{\Gamma}}=8* \frac{\left({\mathbf{P}}^{{\prime}}-0.5\right)*\left(\mathbf{P}-0.5\right)}{m},$$

where **P** is a $m$ x $k$ matrix of strata-specific allele frequencies.

### Approximation of ${\mathbf{A}}^{{\varvec{\Gamma}}}$

Founders defined as unrelated in $\mathbf{A}$ are considered to be related and inbred in ${\mathbf{A}}^{{\varvec{\Gamma}}}$ [12]. Established methods to transfer the information in ${\varvec{\Gamma}}$ to the full relationship matrix of genotyped and non-genotyped animals use ${\varvec{\Gamma}}$ as a kernel and develop ${\mathbf{A}}^{{\varvec{\Gamma}}}$ by uniquely assigning pedigree-founders to one or two metafounders using standard algorithms to set up relationship matrices (e.g., the ‘tabular method’ in [12]). Legarra et al. [12] proposed an approximation of ${\mathbf{A}}^{{\varvec{\Gamma}}}$, by assigning founders to metafounders and tracing contributions based on the pedigree. Since we estimated matrix $\mathbf{Q}$ directly from genomic data, it was not straightforward to assign founders which are typically non-genotyped animals, directly to genomic strata. A simple calculation of matrix ${\mathbf{A}}^{{\varvec{\Gamma}}}$ using standard algorithms was therefore not possible. To investigate whether ADMIXTURE was successful in recovering latent stratification and whether the introduction of this stratification information into ${\mathbf{A}}_{g}$ leads in fact to an improved concordance between conventional and genomic relationships, we calculated an approximation of ${\mathbf{A}}^{{\varvec{\Gamma}}}$ for genotyped animals only.

In our approach to approximate ${\mathbf{A}}^{{\varvec{\Gamma}}}$ we tried to separate the introduction of stratification information into ${\mathbf{A}}_{g}$ from the process of rescaling the relationship matrix to a different genetic base. This stepwise approach elucidates some aspects of the metafounder approach that are not immediately obvious otherwise.

To approximate ${\mathbf{A}}^{{\varvec{\Gamma}}}$, we first calculated a pivotal $\gamma$, representing the overall self-relationship of the pedigree base relative to a hypothetical base of maximum heterozygosity. This pivotal $\gamma$ can for example be directly estimated using matrices ${\mathbf{A}}_{g}$ and $\mathbf{G}$ via [12]

5 $$\gamma =\frac{\overline{\mathbf{G} }-\overline{{\mathbf{A} }_{g}}}{1-\overline{{\mathbf{A} }_{g}}/2},$$

when $\mathbf{G}$ was calculated with base allele frequencies of 0.5. Note that this pivotal $\gamma$ directly provides an estimate of the overall heterozygosity of the pedigree base (${H}_{B}$) given that ${H}_{B}=0.5-0.25\gamma$ (assuming homogeneity and HWE).

If the matrix ${\varvec{\Gamma}}$ of ‘self-relationships’ of metafounder has been calculated as described in (4), its implicit reference is a base of maximum heterozygosity with a base allele frequency of 0.5 (${H}_{X}=0.5$) under HWE. We have indicated this with subscript $X$, using ${{\varvec{\Gamma}}}_{X}$ in the following. Such a ${{\varvec{\Gamma}}}_{X}$ cannot readily be combined with a standard relationship (sub-) matrix ${\mathbf{A}}_{g}$ in an additive manner, since ${\mathbf{A}}_{g}$ is referring to the actual heterozygosity of the pedigree base (${H}_{B}$). Therefore ${{\varvec{\Gamma}}}_{X}$, must be rescaled to refer to the same base as ${\mathbf{A}}_{g}$ by calculating

$${{\varvec{\Gamma}}}_{B}= {P}_{X,B} \left({{\varvec{\Gamma}}}_{X}- 2\right)+ 2=$$

$$\frac{{H}_{X}}{{H}_{B}} \left({{\varvec{\Gamma}}}_{X} -2\right)+ 2=$$

$$\frac{1}{2{H}_{B}} \left({{\varvec{\Gamma}}}_{X}-2\right)+ 2,$$

Matrix ${{\varvec{\Gamma}}}_{B}$ then refers to the same genetic base as matrix ${\mathbf{A}}_{g}$ but it is not in the standard form of a relationship matrix. To achieve this, we calculated

$${{{\varvec{\Gamma}}}_{B}}^{\boldsymbol{*}}=\left(\mathbf{I}-{\mathbf{D}}_{B}\right)+\boldsymbol{ }{{\varvec{\Gamma}}}_{B},$$

where $\mathbf{I}$ is an identity matrix, and ${\mathbf{D}}_{B}$ is a diagonal matrix containing the diagonal elements of ${{\varvec{\Gamma}}}_{B}$ multiplied by 0.5. Stratification information as represented by ${{{\varvec{\Gamma}}}_{B}}^{\boldsymbol{*}}$ is then combined with the standard relationship matrix ${\mathbf{A}}_{g}$ to give an approximate ${\mathbf{A}}_{B}^{{\varvec{\Gamma}}}$ by calculating

6 $${\mathbf{A}}_{B}^{{\varvec{\Gamma}}}\approx {\mathbf{A}}_{g}+ \mathbf{Q}\left({{{\varvec{\Gamma}}}_{B}}^{\boldsymbol{*}}-\mathbf{I}\right){\mathbf{Q}}^{{\prime}},$$

Matrix ${\mathbf{A}}_{B}^{{\varvec{\Gamma}}}$ includes information on stratification but still refers to the heterozygosity of the pedigree base (indicated by subscript $B$). To compare or combine it with a matrix $\mathbf{G}$ calculated referring to a base of maximum heterozygosity it has to be rescaled to finally give ${\mathbf{A}}_{X}^{{\varvec{\Gamma}}}$ or simply ${\mathbf{A}}^{{\varvec{\Gamma}}}$ (in concurrence with [12])

7 $${\mathbf{A}}^{{\varvec{\Gamma}}}\approx 2{H}_{B}\left({\mathbf{A}}_{B}^{{\varvec{\Gamma}}}- 2\right)+ 2,$$

### Evaluation of results

We used several evaluation criteria to test the improvement of the compatibility of the derived ${\mathbf{A}}^{{\varvec{\Gamma}}}$ and $\mathbf{G}$ as compared to the compatibility of ${\mathbf{A}}_{g}$ and $\mathbf{G}$. Legarra et al. [12] recommended the comparison of overall means and mean diagonal values of ${\mathbf{A}}^{{\varvec{\Gamma}}}$ and $\mathbf{G}$ as evaluation criteria. If no difference in overall means exists, this indicates that both matrices refer to the same overall heterozygosity and no further scaling is necessary (pivotal $\gamma$ of zero). For the comparison of means we computed the difference in means of all matrix elements ($\overline{\mathbf{G} }-{\overline{\mathbf{A}} }^{{\varvec{\Gamma}}}$) and the difference in the means of the diagonal elements ($\overline{\text{diag }(\mathbf{\text{G}})}-{\overline{\text{diag }(\mathbf{\text{A}}}}^{{\varvec{\Gamma}}})$). Both criteria have optimum values of 0.

Additionally, we regressed elements of ${\mathbf{A}}^{{\varvec{\Gamma}}}$ on $\mathbf{G}$, and calculated intercept ($a$), slope ($b$) and fit (R^2^) of the regression [3, 42]. Using this form of regression has the advantage of keeping the independent variable constant throughout the optimization process. When evaluating ${\mathbf{A}}^{{\varvec{\Gamma}}}$, R^2^ should be at least as high as for the regression of $\mathbf{A}$ on $\mathbf{G}$. However, the expectations of *a* and *b* are not intuitively obvious. Authors of [42] used a similar regression of standard coancestry coefficients derived from matrix **A** on genomic coancestries derived from covariance like versions of the genomic relationship matrix **G**, similar to those proposed here. They argued that the estimates of intercept and slope in this form of regression should be close to 0 and 1 if **A** (${\mathbf{A}}^{{\varvec{\Gamma}}}$) and $\mathbf{G}$ are properly scaled to same genetic base [42]. They confirmed this expectation by dropping 10,000 unlinked loci through a pedigree of ten discrete generations using uniform founder allele frequencies of 0.5. When introducing linkage in the simulation they found however, that estimated slopes tended to be generally lower than 1, indicating that the dispersion of relationships in **G** with linkage should be larger than the dispersion of corresponding coefficients in **A** [42]. Transferring this result to our situation we argue that an upper limit of 1 for the slope should be a reasonable criterion in the evaluation of the dispersion in ${\mathbf{A}}^{{\varvec{\Gamma}}}$.

Principal components analysis (PCA) of SNP-genotypes is an established method to uncover and visualize population stratification [46]. PCA was performed using the eigen() function in R v.3.5.2 [47] on ${\mathbf{A}}^{{\varvec{\Gamma}}}$ and $\mathbf{G}$, respectively, and the first two principal components were plotted with the ggplot2 package [48] to visualize the effect of an increased number of strata ($k$) on the composition of ${\mathbf{A}}^{{\varvec{\Gamma}}}$. In all plots, eigenvectors were multiplied by their respective eigenvalues to reflect the proportion of total variance associated with the respective principal component.

### Dataset

To investigate the feasibility of our approach we used genotype and pedigree data of the Brown Swiss breed. Earlier studies on Brown Swiss showed that this population exhibits a considerable degree of stratification due to its breeding history [36, 37, 49]. In short, the Brown Swiss breed originates from Switzerland. Export of breeding animals began in the nineteenth century [50]. Some of these animals founded the US Brown Swiss population [51]. In Europe and the US different breeding goals were pursued (multipurpose vs. dairy focused). Starting in the 1960s, US semen was used extensively in the European Brown Swiss population to adapt the breed to the changing demands. On the other hand, there was only a marginal contribution of European populations to the US population after the initial phase in the nineteenth century. In the 1980s Original Braunvieh (OBV) herdbooks were established to preserve the original multipurpose type of the breed. These herdbooks excluded the registration of animals with US contributions [52]. Today most Brown Swiss animals in Europe have a very high proportion of the US breed and coexist with small populations of OBV.

Analyses were performed on the genotypes used in the joint Single-Step breeding value estimation of the German and Austrian Brown Swiss population in April 2022. Additionally, 1180 OBV animals with genotypes were included in this set. These are currently not considered in the routine genetic evaluation. Declaration of OBV status differs slightly between countries, most OBV genotypes come from Switzerland (1114). In total, 85,249 genotypes were available. These include historic bulls born before 2000, bull dams and selection candidates born from 2000 to 2010 and genotypes of cows and selection candidates from birthyear 2010 onwards. Of the 85,249 genotyped animals, 100 had a missing sire, 363 a missing dam. There were no animals with both parents missing. In 1630 cases there was only one genotyped offspring per sire, three sires had more than 1200 genotyped offspring each. The standard pedigree used in routine breeding value estimation for these 85,249 animals consisted of 316,579 animals, which are the ancestors of the genotyped animals. Table 1 displays the distribution of the genotyped animals across countries of origin and sex. In the definition of country of origin, animals from Germany and Austria were grouped into ‘DEA’ and animals from USA and Canada were grouped into ‘USACAN’. Apart from those groups, Switzerland (CHE) and Italy (ITA) were countries of origin with larger animal numbers. The remaining eleven countries were grouped into ‘OTHER’. Foreign (i.e., not DEA) genotypes mainly came from AI-bulls, whereas for the DEA population most of the genotypes were from cows. Genotypes were predominantly available for recent birth years, only a few bulls with birth years before 2000 were genotyped. A similar structure could be observed for the OBV group (see Additional file 2 Table S1). From the Intergenomics initiative, genotypes of foreign ancestors of DEA bulls were in many cases available. The available genotypes were checked for parental assignment and corrected, if necessary. Because of ADMIXTURE’s sensitivity to familial structure, additional samples of genotyped animals were constructed, by a simple rule-based approach:

- For every paternal half-sib group select one member
- For every maternal half-sib group select one member
- For every animal with both parents genotyped, remove its offspring
- Keep all animals with unknown parents

**Table 1:** Number of genotyped animals for each sample and distribution across country of origin

	N	Sex	CHE	DEA	ITA	OBV	OTHER	USACAN
S1	85,249	Male	4929	22,166	2530	1177	1721	2131
		Female	18	50,505	5	3	49	15
S2	4152	Male	598	900	252	455	186	228
		Female	0	1530	0	1	2	0
S3	4150	Male	468	759	183	445	155	170
		Female	1	1963	0	1	5	0
S4	4152	Male	261	1047	133	63	114	100
		Female	0	2428	0	0	3	2

*N* Number of animals in sample, assignment was done by country of origin, *CHE* Switzerland, *DEA* Germany and Austria, *ITA* Italy, *OBV* Animals recorded as Original Braunvieh, *USACAN* United States of America and Canada, *OTHER* Other country of origin

Following these rules, two samples were drawn, one selecting only the offspring with the highest call-rate (S2), the other one selecting one random member of each group (S3). This resulted in 4152 and 4150 animals for S2 and S3, respectively. To test the effect of sample size on the results, 4152 animals (S4) were randomly selected from all genotyped animals. The structure of these additional samples is also outlined in Table 1.

In routine genotyping of DEA animals, an Illumina Bovine BeadChip (Illumina Inc., San Diego, CA) with approximately 43K SNPs customized for the DEA population was used. From animals genotyped with other chips, only these 43K markers were retained. For all animals, after the initial edits (i.e., exclusion of markers with call-rate < 0.95, minor allele frequency < 0.01 or redundancy with another locus), 42,384 SNPs markers remained for the computation of $\mathbf{G}$. For the estimation of allele frequencies only annotated SNPs were used (41,950). ADMIXTURE assumes linkage equilibrium between SNPs [35]. Calus et al. [53] reported a loss of accuracy in ADMIXTURE results when SNP selection is either too stringent or lenient. We considered only every other SNP on each chromosome, to reduce LD while retaining enough SNPs to achieve accurate estimates, resulting in 20,983 SNPs in the ADMIXUTRE analyses.

## Results

### Comparison of initial ${\mathbf{A}}_{{\varvec{g}}}$ and $\mathbf{G}$

Using the submatrix of genotyped animals taken from a standard numerator relationship matrix based on the existing pedigree (${\mathbf{A}}_{g}$) the pivotal $\gamma$ was calculated to be 0.694 corresponding to an average heterozygosity of the pedigree base of $0.5-0.25\gamma =0.327$ (Table 2). In the initial situation $\mathbf{G}$ and ${\mathbf{A}}_{g}$ therefore refer to different average base heterozygosities (0.5 vs. 0.327) and are not directly comparable. This is also indicated by a highly negative value for the intercept when regressing ${\mathbf{A}}_{g}$ on $\mathbf{G}$ (Table 2).

**Table 2:** Results of the regression of ${\mathbf{A}}_{g}$ and ${\mathbf{A}}^{{\varvec{\upgamma}}}$ on $\mathbf{G}$

Matrix	a	b	R^2^	$\overline{G} - \overline{A}$	$\overline{diag(G)}- \overline{diag(\text{A})}$	Pivotal γ
$\text{G}$						0.694
${\text{A}}_{g}$	− 0.489	0.780	0.647	0.657	0.330	
${\text{A}}^{\upgamma }$	0.375	0.509	0.647	0	0	

a = intercept, b = slope of the regression, R^2^ = fit of the regression, $\text{G}$ = genomic relationship matrix, ${\text{A}}_{g}$=submatrix of genotyped animals of the numerator relationship matrix, ${\text{A}}^{\text{y}}={\text{A}}_{g}$ rescaled by γ, $\overline{\text{G}}-\overline{\text{A}}$=Difference of means of respective matrices, $\overline{\text{diag }\left(\text{G}\right)}- \overline{\text{diag}(\text{A})}$=Difference of the means of the diagonals of respective matrices

**Fig. 1:**
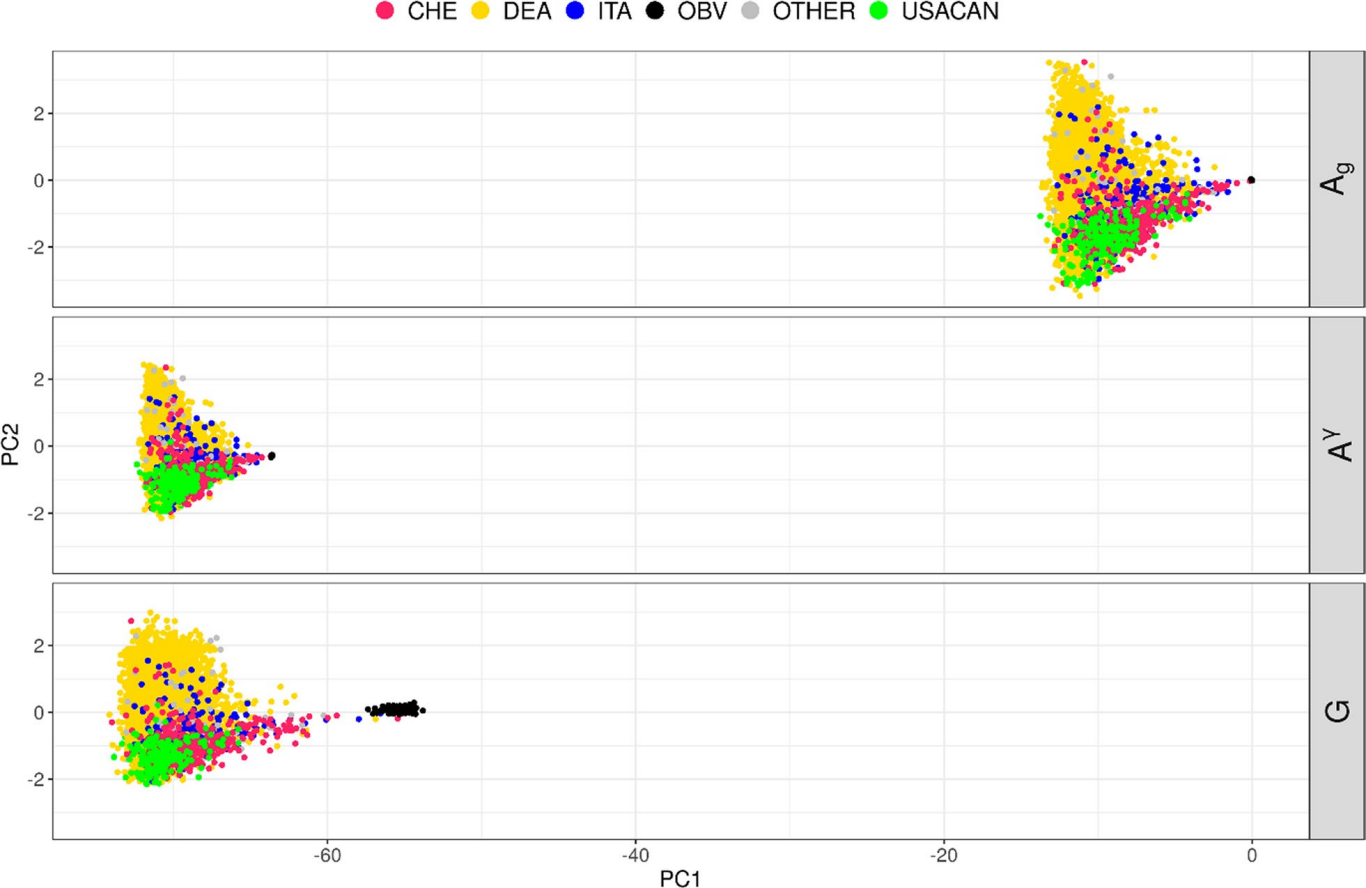
PCA Plot of ${\text{A}}_{g}$, ${\text{A}}^{\upgamma }$ and $\text{G}$ matrix. All comparisons are done based on a random subsample of ~ 10% of the genotyped animals. Animals are colored according to country of origin taken from ISO number, except for registered OBV which are all in black

When ${\mathbf{A}}_{g}$ was scaled by the pivotal $\gamma$ to match $\mathbf{G}$, the mean and mean-diagonal values of resulting ${\mathbf{A}}^{\upgamma }$ and $\mathbf{G}$ were identical. Values for slope and R^2^ were identical to the initial ${\mathbf{A}}_{g}$, but the intercept was still comparably large. Comparing PCA plots of ${\mathbf{A}}^{\upgamma }$ and $\mathbf{G}$ confirmed both to be on a similar scale, as opposed to the initial situation with ${\mathbf{A}}_{g}$ (Fig. 1). Differences between the numerator relationship matrix and the genomic relationship matrix are visible in Fig. 1. Along the first principal component (PC), ${\mathbf{A}}^{\upgamma }$ and ${\mathbf{A}}_{g}$ underestimated the distance between OBV and Brown Swiss subpopulations which is apparent in the PCA of $\mathbf{G}$. Information on variation within the OBV cluster was also missing from ${\mathbf{A}}^{\upgamma }$ and ${\mathbf{A}}_{g}$. All three matrices (${\mathbf{A}}_{g}$, ${\mathbf{A}}^{\upgamma }$ and $\mathbf{G}$) showed a gradient along PC2 differentiating USACAN and OTHER from DEA.

### Detecting stratification and ${\varvec{\Gamma}}$

**Fig. 2:**
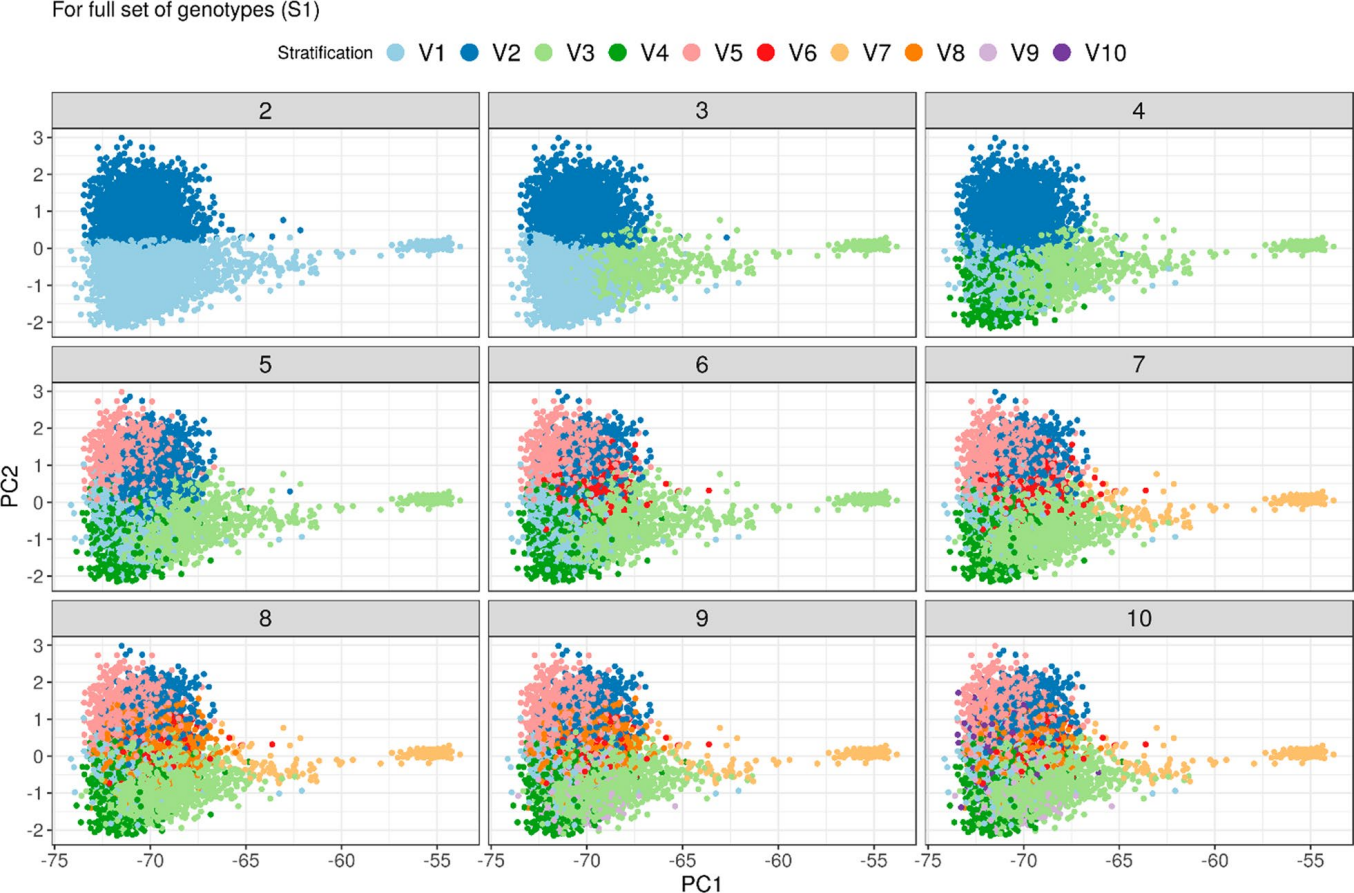
PCA Plot of $\mathbf{G}$, color according to identified stratification for ADMIXTURE run in full set of genotypes (S1)

ADMIXTURE assigns anonymous stratifications based on the input data. To interpret ADMIXTURE’s strategy of assignment, animals in PCA plots of $\mathbf{G}$ were colored according to the identified dominant stratum (V1 to V10) for $k$ = 2 to 10 (e.g., an animal with at least 33% assignment to stratum V1 for k = 3, is assigned to V1) (Figs. 2 and 4).

#### Full sample (S1)

For S1 and $k$ = 2, ADMIXTURE detected the groups of DEA and USACAN as the most important sources of stratification. Only for $k$ ≥ 7 OBV was detected as a relevant stratum (Fig. 2).

**Fig. 3:**
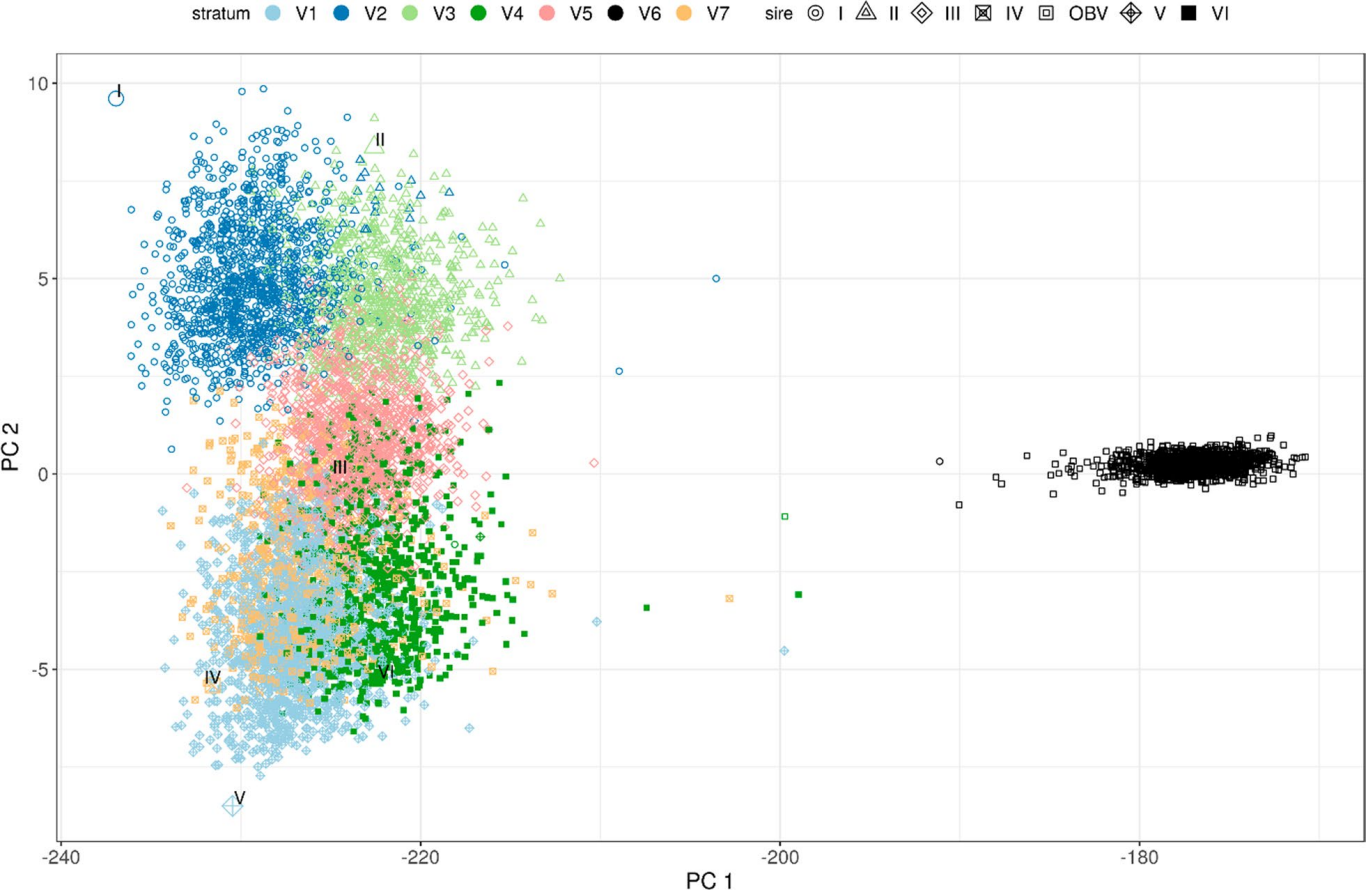
PCA of large half-sib families corresponding to stratification identified by ADMIXTURE in the full set of genotypes (S1). Separable groups within modern Brown Swiss relate to specific sires. Shape indicates parentage of a specific sire. Larger Shapes indicate the specific sires themselves. Registered OBV are all one shape. Color is according to assigned stratification of specific animal

For illustration purposes the matrix ${\varvec{\Gamma}}$ for $k$ = 7 was rescaled to the heterozygosity of the pedigree base using the pivotal $\gamma$ of 0.694 and presented in the form of a conventional relationship matrix (Table 3) (for details see "[Sec Sec8]" in "Methods" section). Stratum 6 showed large negative relationships (range – 0.223 to – 0.190) to all other strata and a diagonal value less than 1 (0.963). All other strata had a diagonal value larger than 1 and relationships between strata ranged from – 0.005 (3 to 7) to 0.144 (1 to 4). Closer inspection of the strata showed that V6 corresponded to the OBV group while all other strata comprised large half-sib families (Fig. 3). Sires V and VI were important US sires, and although Sire IV was registered with a Swiss ISO code, its pedigree contained many US animals. Sires I, II, and III were sires registered in Germany. The 10 most influential sires in our dataset had more than 800 offspring each. Sires V, III, and I each had more than 1200 offspring in the data. For PC3 and PC4 the separation of US-influenced half-sib groups was clearer (See Additional file 1 Fig. S1).

**Table 3:** ${\varvec{\Gamma}}$ rescaled using pivotal ${\varvec{\gamma}}$ and displayed as a relationship matrix, for k = 7 for full set of genotypes (S1)

	Stratum 1	Stratum 2	Stratum 3	Stratum 4	Stratum 5	Stratum 6	Stratum 7
Stratum 1	1.208						
Stratum 2	0.074	1.195					
Stratum 3	0.037	0.062	1.170				
Stratum 4	0.144	0.132	0.073	1.145			
Stratum 5	0.089	0.100	0.049	0.132	1.164		
Stratum 6	− 0.218	− 0.190	− 0.202	− 0.212	− 0.223	0.963	
Stratum 7	0.108	0.061	− 0.005	0.128	0.094	− 0.205	1.232

For the sake of readability only the lower triangular matrix is displayed

#### Reduced relationships sample (S2)

**Fig. 4:**
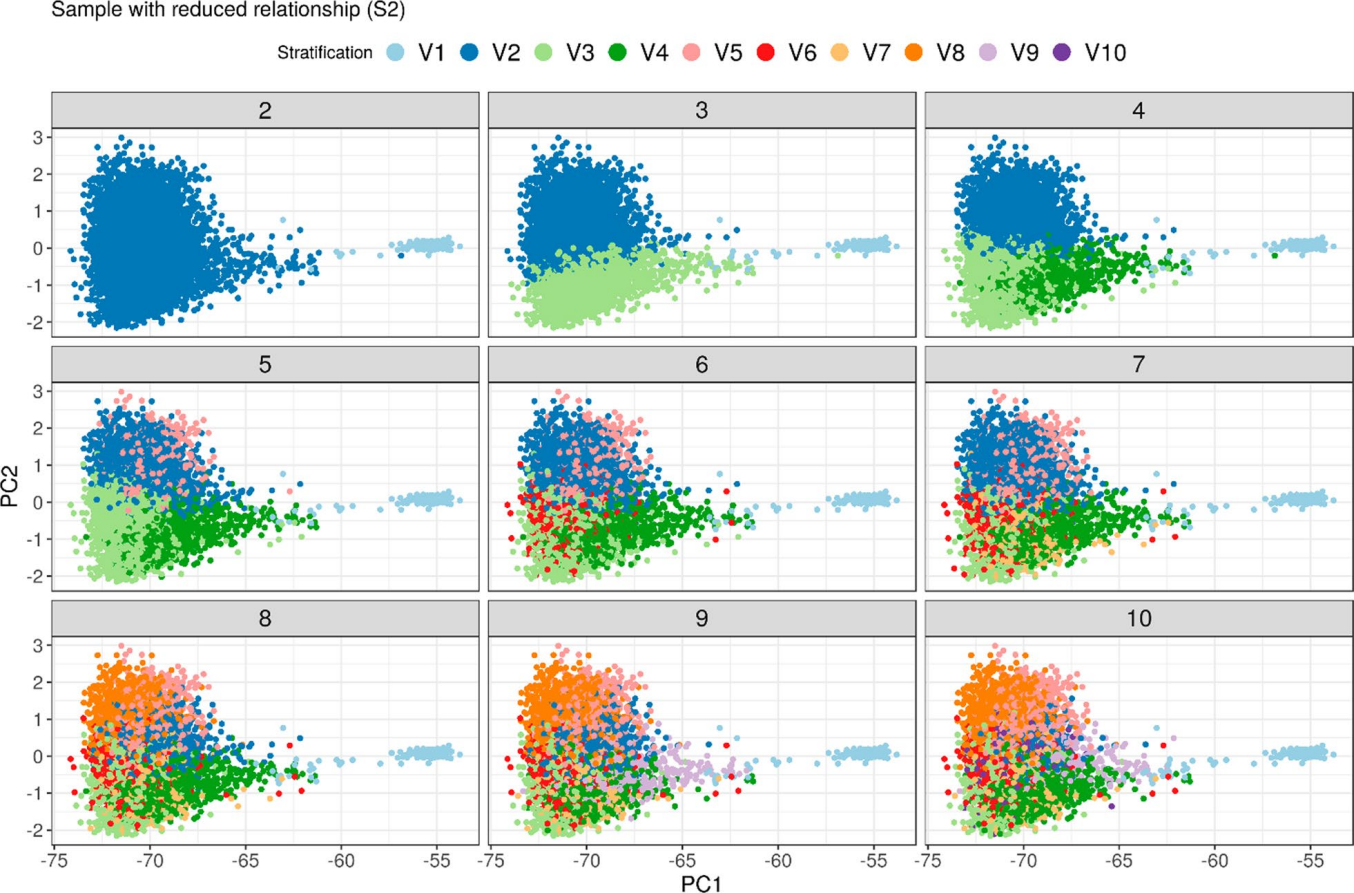
PCA plot of $\mathbf{G}$, color according to identified stratification for ADMIXTURE run in a sample with reduced relationship (S2)

ADMIXTURE is sensitive to familial structures in the data. This was described by several authors [25, 26, 53] and can also be seen from the results of the analysis of S1 which showed that many strata correspond to paternal half-sib families. This makes it more difficult to detect the underlying structure of the base population. Information on familial structures on the other hand was in many cases available from pedigree data. Therefore, we reduced the relationships between genotypes used in the analysis by sampling unrelated individuals (S2). In this sample ADMIXTURE identified OBV as a relevant stratification already from $k$ = 2 on, splitting the population along the x-axis as expected (Fig. 4). Along the y-axis, $k$ = 3 shows a first separation, roughly corresponding to DEA and USACAN subpopulations.

### Adjusting ${\mathbf{A}}_{{\varvec{g}}}$ to $\mathbf{G}$: evaluation of ${\mathbf{A}}^{{\varvec{\Gamma}}}$

Results of the process of adjusting ${\mathbf{A}}_{g}$ to $\mathbf{G}$ by increasing $k$ from 2 to 40 for the full (S1) and reduced sample (S2) are presented in Tables 4 and 5. Results for additional control samples S3 (without half-sib structure) and S4 (with half-sib structure) are presented in Additional file 2 Tables S2 and S3, respectively.

**Table 4:** Results for full set of genotypes (S1)

k	a	b	R^2^	$\overline{G }- \overline{{A }^{\Gamma }}$	$\overline{diag\left(G\right)}- \overline{diag({A}^{\Gamma })}$
2	0.340	0.530	0.637	0.019	0.015
3	0.250	0.668	0.690	0.003	− 0.001
4	0.245	0.674	0.689	0.004	− 0.002
5	0.218	0.710	0.701	0.004	− 0.005
6	0.196	0.738	0.706	0.005	− 0.006
7	− 0.028	1.087	0.807	− 0.038	− 0.049
8	− 0.058	1.128	0.808	− 0.040	− 0.054
9	− 0.078	1.150	0.808	− 0.037	− 0.053
10	− 0.074	1.141	0.808	− 0.034	− 0.051
11	− 0.076	1.139	0.810	− 0.030	− 0.050
12	− 0.069	1.123	0.807	− 0.025	− 0.047
13	− 0.065	1.115	0.804	− 0.023	− 0.046
14	− 0.068	1.114	0.807	− 0.019	− 0.044
15	− 0.091	1.147	0.807	− 0.022	− 0.048
16	− 0.068	1.108	0.808	− 0.014	− 0.042
17	− 0.086	1.133	0.804	− 0.017	− 0.045
18	− 0.082	1.124	0.806	− 0.012	− 0.042
19	− 0.087	1.126	0.808	− 0.010	− 0.041
20	− 0.082	1.117	0.806	− 0.008	− 0.040
21	− 0.095	1.134	0.809	− 0.008	− 0.040
22	− 0.081	1.113	0.804	− 0.005	− 0.039
23	− 0.083	1.115	0.805	− 0.004	− 0.038
24	− 0.087	1.117	0.806	− 0.003	− 0.038
25	− 0.090	1.120	0.806	− 0.001	− 0.037
26	− 0.087	1.113	0.807	0.001	− 0.036
27	− 0.094	1.122	0.805	0.001	− 0.036
35	− 0.093	1.107	0.801	0.012	− 0.032
40	− 0.100	1.112	0.803	0.015	− 0.031

k = number of stratifications considered, a = intercept, b = slope of the regression, R^2^ = fit of the regression, $\text{G}$=genomic relationship matrix, ${{A}}^{\Gamma }$=numerator relationship matrix amended by stratification information, $\overline{\text{G} }- \overline{{\text{A} }^{\Gamma }}$=Difference of means of both matrices, $\overline{\text{diag }(\text{G})}- \overline{\text{diag}({\text{A}}^{\Gamma })}$=Difference of the means of the diagonals of both matrices

**Table 5:** Results for sample with reduced relationship (S2)

k	a	b	R^2^	$\overline{G }- \overline{{A }^{\Gamma }}$	$\overline{diag({G})}- \overline{diag({A}^{\Gamma })}$
2	− 0.032	1.068	0.802	− 0.020	− 0.025
3	− 0.021	1.074	0.783	− 0.035	− 0.037
4	− 0.050	1.108	0.793	− 0.033	− 0.039
5	− 0.055	1.111	0.795	− 0.030	− 0.038
6	− 0.056	1.109	0.798	− 0.027	− 0.037
7	− 0.058	1.110	0.798	− 0.026	− 0.037
8	− 0.072	1.126	0.802	− 0.024	− 0.038
9	− 0.070	1.118	0.801	− 0.020	− 0.035
10	− 0.068	1.109	0.805	− 0.015	− 0.032
11	− 0.069	1.106	0.809	− 0.012	− 0.030
12	− 0.073	1.109	0.809	− 0.011	− 0.029
13	− 0.068	1.099	0.807	− 0.008	− 0.026
14	− 0.068	1.095	0.809	− 0.005	− 0.024
15	− 0.069	1.093	0.811	− 0.002	− 0.022
16	− 0.066	1.084	0.811	0.002	− 0.019
17	− 0.065	1.079	0.811	0.005	− 0.017
18	− 0.061	1.072	0.812	0.006	− 0.016
19	− 0.061	1.069	0.812	0.008	− 0.014
20	− 0.065	1.072	0.812	0.010	− 0.012
21	− 0.062	1.064	0.811	0.013	− 0.010
22	− 0.059	1.059	0.812	0.014	− 0.009
23	− 0.045	1.038	0.818	0.015	− 0.008
24	− 0.020	0.998	0.817	0.021	− 0.003
25	− 0.020	0.995	0.817	0.024	− 0.001
26	− 0.018	0.989	0.815	0.027	0.003
27	− 0.019	0.986	0.815	0.029	0.004
28	− 0.016	0.981	0.814	0.031	0.006
29	− 0.015	0.975	0.813	0.034	0.008
30	− 0.029	0.992	0.813	0.035	0.011
31	− 0.028	0.989	0.814	0.037	0.012
32	− 0.035	0.996	0.813	0.039	0.014
33	− 0.028	0.982	0.812	0.042	0.016
34	− 0.027	0.978	0.811	0.044	0.018
35	− 0.027	0.975	0.809	0.045	0.020
36	− 0.025	0.970	0.806	0.048	0.022
37	− 0.026	0.969	0.810	0.050	0.024
38	− 0.027	0.968	0.808	0.051	0.025
39	− 0.027	0.965	0.808	0.054	0.027
40	− 0.026	0.960	0.806	0.056	0.029

k = number of stratifications considered, a = intercept, b = slope of the regression, R^2^ = fit of the regression, $\text{G}$=genomic relationship matrix, ${\text{A}}^{\Gamma }$=numerator relationship matrix amended by stratification information, $\overline{\text{G} }- \overline{{\text{A} }^{\Gamma }}$=Difference of means of both matrices, $\overline{\text{diag }\left(\text{G}\right)}- \overline{\text{diag }({\text{A}}^{\Gamma })}$=Difference of the means of the diagonals of both matrices

#### Full sample (S1)

**Fig. 5:**
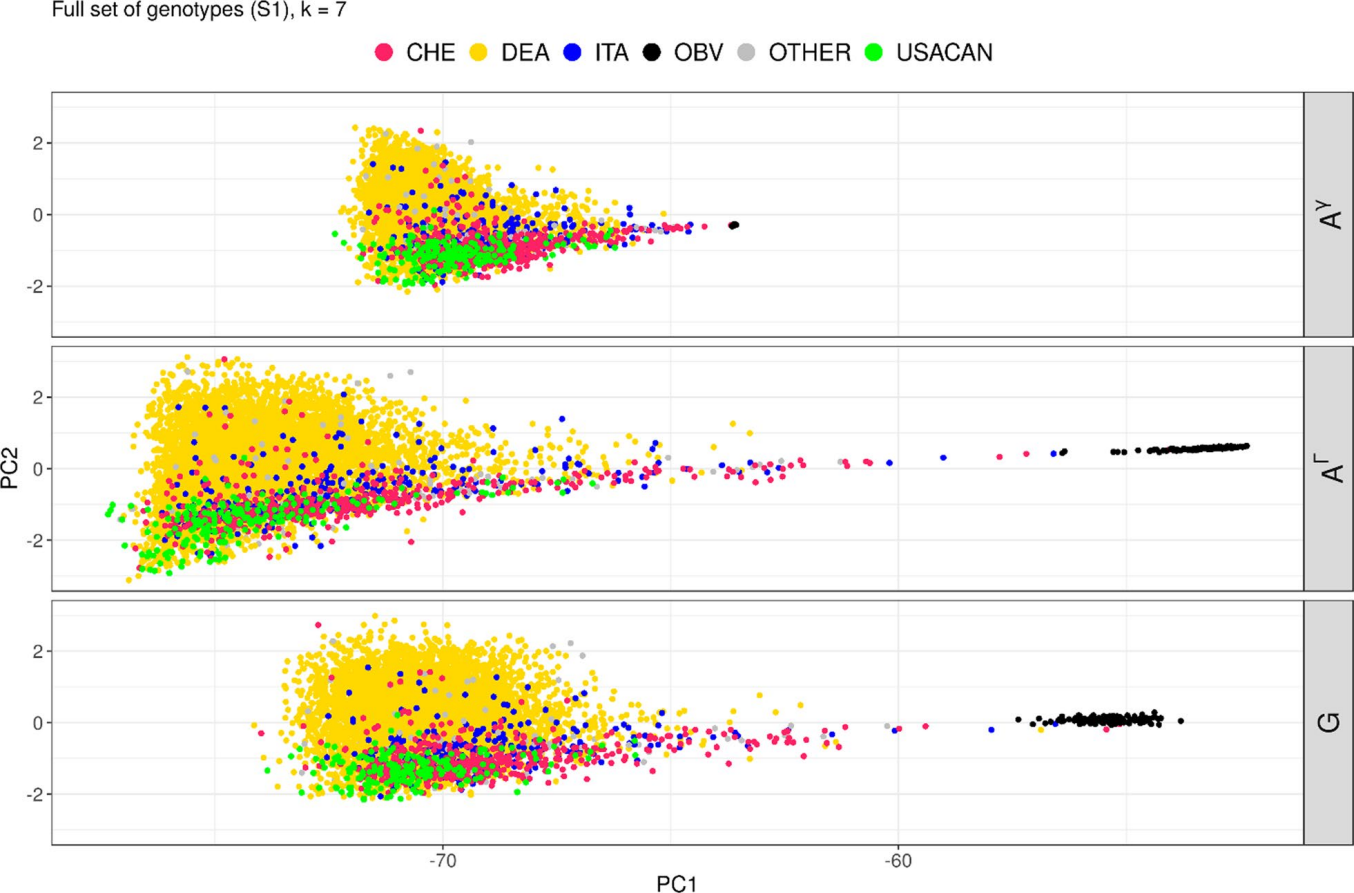
PCA of ${\mathbf{A}}_{g}$, $\mathbf{G}$ and ${\mathbf{A}}^{{\varvec{\Gamma}}}$ from the full set of genotypes (S1) for k = 7. ${\mathbf{A}}^{{\varvec{\Gamma}}}$ shows more variation between Brown Swiss and OBV, and within Brown Swiss cluster than seen in $\mathbf{G}$

For S1 a strong increase in R^2^ (0.706 to 0.807) was observed from $k$ = 6 to $k$ = 7, and $k$ = 7 was the first $k$ to identify OBV as a distinct stratum (Fig. 2). Beyond $k$ = 7, R^2^ fluctuated around 0.810 but estimates of slopes indicated an increasing overdispersion of ${\mathbf{A}}^{{\varvec{\Gamma}}}$ between $k$ = 7 and $k$ = 11 and in addition, larger mean differences (globally and diagonally) were observed. For $k$ ≥ 11, R^2^ remained high with only minimal fluctuations, and no further improvements in $a$ or $b$ were observed. Overdispersion remained more or less constant. However, for $k$ between 20 and 30 the global mean difference reached values very close to zero (pivotal gamma of zero) indicating that the adjustment with respect to global heterozygosity might be ideal somewhere in this range. As indicated by a $b$-value larger than 1, overdispersion for $k$ = 7 was also visible in the PCA plot (Fig. 5).

#### Reduced relationships sample (S2)

**Fig. 6:**
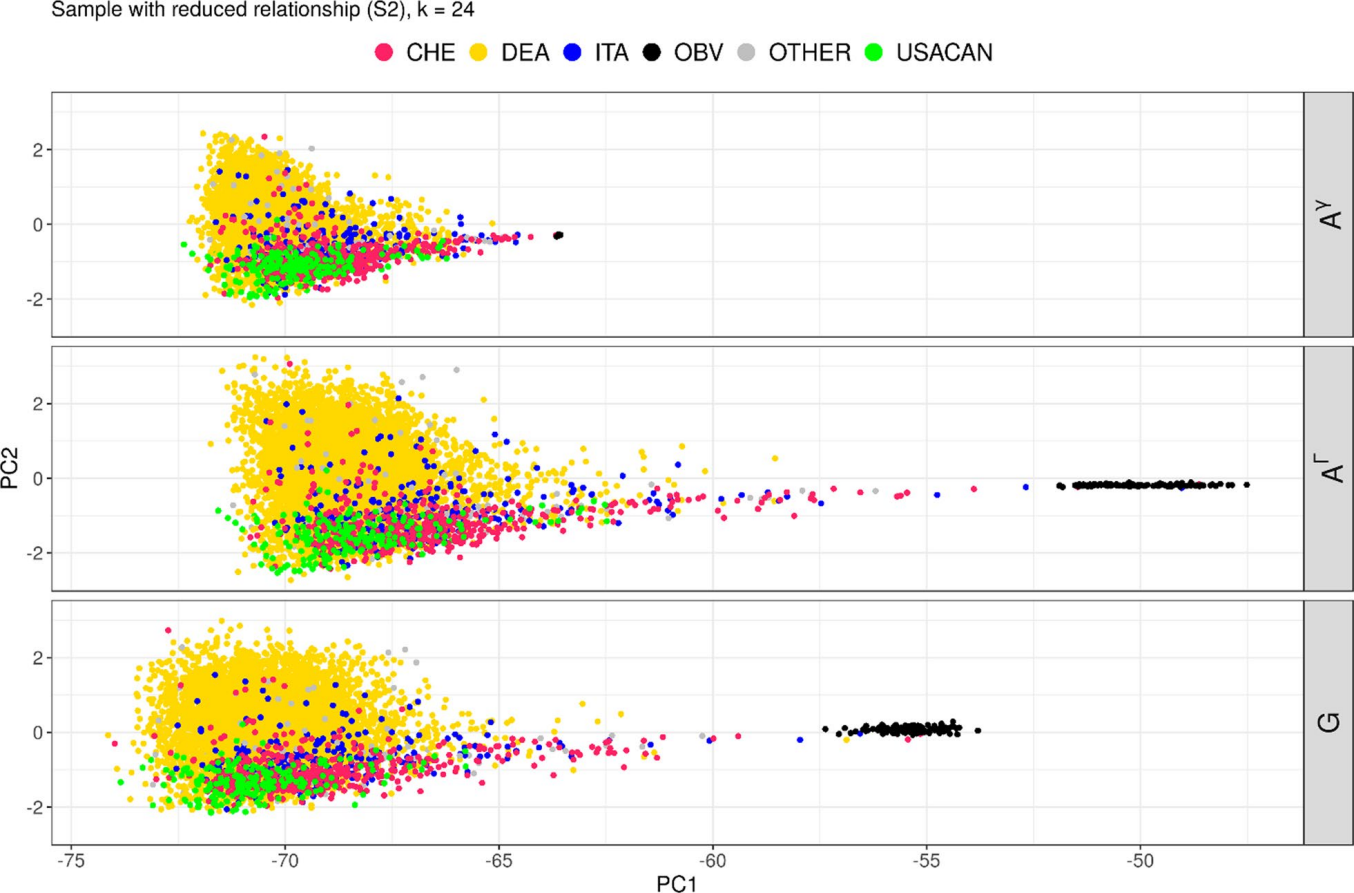
PCA ${\mathbf{A}}_{g}$, $\mathbf{G}$, ${\mathbf{A}}^{{\varvec{\Gamma}}}$ for ADMXITURE a sample with reduced relationship (S2) and k = 24**.** Shift of ${\mathbf{A}}^{{\varvec{\Gamma}}}$ to the right, somewhat larger variaton in OBV group, but overall distance between OBV and Brown Swiss is closer to $\mathbf{G}$ than estimates for S1

For *k* = 2 in sample S2 all parameters showed a much better fit than in S1. Only for *k* ≥ 10 R^2^ increased to values greater than 0.802. Global mean differences were at a minimum for $k$ = 15 and $k$ = 16, but values for *a* and *b* were not within optimum range. The investigation sequence revealed a relatively clear approach to a maximum R^2^ that was found for $k$ = 23 to 25 with a value of 0.818 which was noticeably larger than the maximum value found in sample S1. The estimated slope for $k$ = 24 to 25 was below 1, with intercepts and mean differences acceptably small. The plot of PC for $k$ = 24 overall showed a good agreement with a slight shift of ${\mathbf{A}}^{{\varvec{\Gamma}}}$ to the right as compared to ${\mathbf{A}}_{g}$ and $\mathbf{G}$ which is a consequence of the slightly positive mean difference (Fig. 6 and Table 5). The OBV group showed a slightly larger spread than observed in $\mathbf{G}$.

#### Additional samples (S3 and S4)

To investigate the effect of our sampling strategy, two additional samples were drawn (for details see ‘Dataset’ in Methods). For computational reasons ADMIXTURE analysis on S3 and S4 was performed only for *k* = (2–10, 15, 20–25, 30, 35, 40). Results for S3 were similar to results obtained from S2. For $k$ = 24 values close to optimum were found, confirming the results from analyses of S2. Evaluation criteria for S3 showed a very similar trend in approaching an optimal value as observed for S2 (See Additional file 2 Table S2). Results of S4 were comparable to those obtained from S1. When OBV was identified as a distinct stratum, there was an increase in R^2^ (for $k$ = 8, 0.721 vs. 0.809). Maximum R^2^ was found for $k$ = 22, values for intercept and slope were close to optimal for $k$ = 8, although a $b$ of 1.033 already indicated a slight overdispersion. As observed with S1, R^2^ might be better for additional $k$, but intercept and slope indicated an increase in overdispersion in ${\mathbf{A}}^{{\varvec{\Gamma}}}$ (See Additional file 2 Table S3). Overall, an optimal range for $k$ could be identified consistently for the ‘unrelated’ samples S2 and S3, since the optimum values for intercept, slope and R^2^ occurred in the same range of $k$. Analysis of samples S1 and S4 considering all evaluation criteria gave no conclusive range for the optimal number of strata. An important difference between S2 and S3 as compared to S1 and S4 was that R^2^ for S2 and S3 increased towards a stable plateau before it began to decrease, whereas S1 and S4 both showed a noticeable increase when OBV was detected as a distinct stratum, followed by inconclusive fluctuations in R^2^.

## Discussion

Our study was performed on genotypes from Brown Swiss animals to test an approach to improve the compatibility of ${\mathbf{A}}_{g}$ and $\mathbf{G}$ by identifying present stratifications in the population using software for population structure analysis and by transferring this information to ${\mathbf{A}}_{g}$ using metafounder methodology. We showed that close relationships, which are common in populations under selection, lead to unexpected results from population structure analysis using ADMIXTURE. Nevertheless, identifying anonymous stratifications and transferring this information to ${\mathbf{A}}_{g}$ using Legarra et al.’s [12] approach and the presented alternative, yielded considerable improvements in the compatibility of ${\mathbf{A}}^{{\varvec{\Gamma}}}$ and $\mathbf{G}$ as indicated by the evaluation criteria.

### Detection of population stratification

Initial PCA confirmed a subdivision within the European Brown Swiss population already described by authors from our group [36, 37]. This subdivision into OBV, DEA, and USACAN clusters could be detected in the ${\mathbf{A}}_{g}$ and $\mathbf{G}$ matrices. However, ${\mathbf{A}}_{g}$ lacked information on variation within the OBV group and underestimated the distance between the modern Brown Swiss population and OBV (Fig. 1).

ADMIXTURE frequently identified half-sib groups as strata when analysis was run on a typical cattle breeding population (S1). Because of the implemented maximum likelihood model, a small error in assignment to a specific stratum for many animals may have more impact than a large error for only few animals. This seemed evident by the order of detected stratifications in S1, where the OBV group was identified only for $k$ ≥ 7 ($k$ ≥ 8 in S4). This indicates that, given the number of genotypes in the various groups, relationships and gradients of drift within the modern Brown Swiss population had stronger influence on the discriminant statistics of ADMIXTURE than the graphically more intuitive separation between modern Brown Swiss and OBV. In total, only 1180 animals were registered as OBV, whereas the three most influential Brown Swiss sires have > 1200 offspring each. The challenge to account for familial structure in ADMIXTURE analysis has been addressed by other authors [25, 26]. In preliminary tests we found the KING approach as suggested by Manichaikul et al. [26], where kinship coefficients are inferred while considering population structure, not suitable for our objective.

Our simple approach where we selected one individual from each half-sib family was easy to implement but interpretation is a challenge, because the sampling changed also the relative importance of the OBV group in our data. We observed that OBV was detected much earlier than in the full dataset, but it cannot be excluded that this is due to the relatively larger size of OBV in S2. However, given the fact that only one genotype per half-sib group was included, it seems obvious that OBV will be detected already for a smaller *k*. The results for S3 were consistent with those from S2. However, due to the familial structure of the data 2366 out of 4152 animals were included in both samples. The importance of the familial structure can also be concluded from the comparison of the results of S1 and S4 which showed similar tendencies despite the large difference in sample size. We conclude that sampling one animal per half-sib family is preferrable to the analysis of the whole dataset, because there is less redundancy between the identified strata and the information contained in ${\mathbf{A}}_{g}$.

ADMIXTURE’s CV-option was not able to detect an optimal value of $k$, as indicated by a clear minimum, for any of the four samples. This trend was also observed by Decker et al. [24] in an analysis of the world cattle population. A steadily decreasing CV-criterion implied that ADMIXTURE continued to find additional stratification in the population. This was most likely due to groups of animals with close relationships in the sample. Even when large half-sib groups were removed (S2 and S3), the remaining animals shared some degree of relationship typical for a modern breeding population under selection.

### Regression

ADMIXTURE only provided rows in $\mathbf{Q}$ for animals, which were part of the ADMIXTURE analysis. When the sample size was reduced (as in S2, S3, and S4), rows in $\mathbf{Q}$ from ADMIXTURE were only available for the 4152 animals in the respective samples. A regression analysis has been successfully applied to estimate genomic breed contributions in scenarios, where allele frequencies of base groups were available [39, 54]. Since ADMIXTURE also provided a matrix ${\mathbf{P}}_{\text{A}}$, with allele frequencies for each stratum, the two approaches can be combined to expand $\mathbf{Q}$ to comprise all genotyped animals. A beneficial side effect of a smaller sample size was the reduction in computing time for ADMIXTURE. Additional investigations showed that estimates from ADMIXTURE $\mathbf{Q}$ for S2 to S4 and estimates from regression $\mathbf{Q}$ for these animals based on the respective ${\mathbf{P}}_{\text{A}}$ show only small differences in assignments (results not shown). The combination of ADMIXTURE and regression could also be helpful for situations where genotypes are constantly added to the data pool, as it is common for genomic breeding value estimation systems in dairy cattle, because it circumvents the computation of all strata every time an evaluation is conducted.

The inclusion of an intercept in the regression of genotypes on strata-allele-frequencies could be used as a quality control. According to Chiang et al. [38] a non-zero intercept can be interpreted as an indication of missing strata in the analysis (value for $k$ too low). For all the investigated samples, animals with an intercept > 0.1 were found. For each additional $k$, less animals showed an intercept > 0.1, but even for $k$ = 40, a small group of 57 to 89 (S2: 57, S3: 64, S4: 89) animals showed an intercept > 0.1. However, our intention was not to detect every possible stratification in the data, but to identify stratification that was not already captured by pedigree data (${\mathbf{A}}_{g}$) and that could be a source of inconsistencies between ${\mathbf{A}}_{g}$ and **G**.

### Metafounder concept

**Fig. 7:**
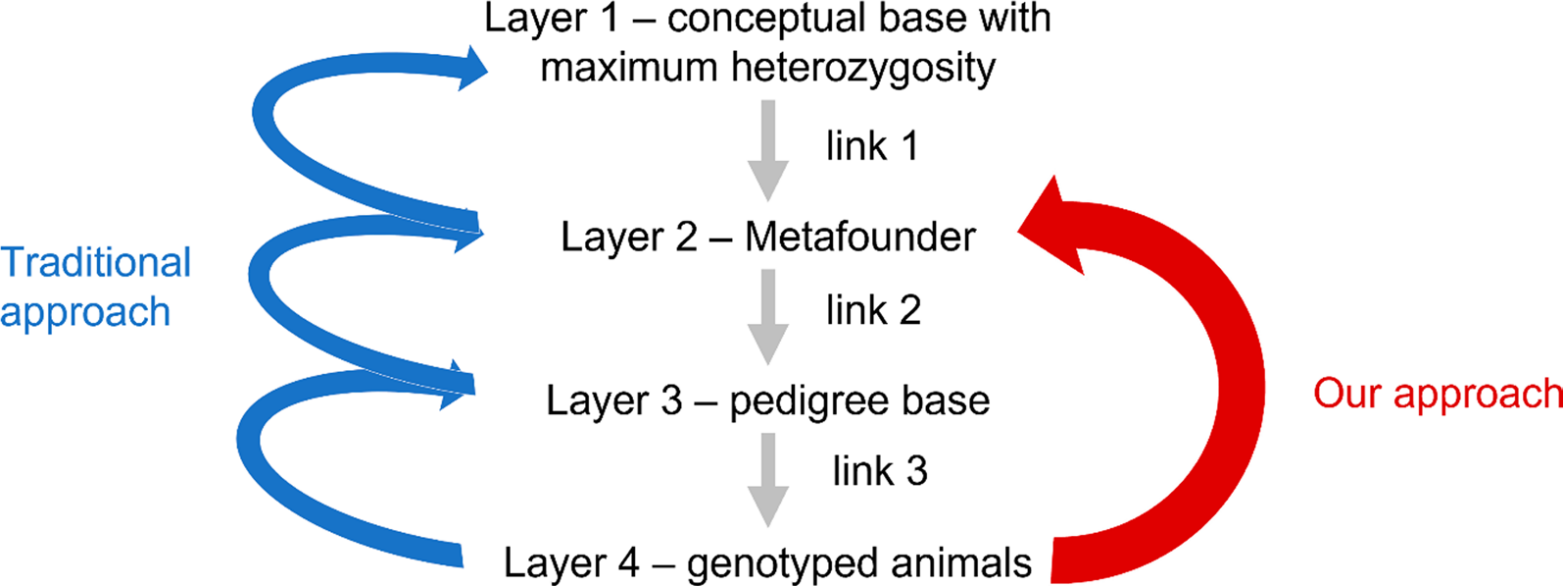
Visualization of Metafounder concept – revisited. In the original Metafounder concept, all layers and the connections between them are relevant (in blue). In our approach we go directly from Layer 4 to Layer 2 (red arrow), skipping Layer 3, the pedigree base

The Metafounder concept can be visualized by four layers and the links between them (Fig. 7). The first layer is a hypothetical unrelated base population with maximum heterozygosity. This base is connected to the layer of metafounders (Layer 2) which is also a conceptual one. Metafounder allele frequencies are necessarily different from the base allele frequencies which implies that metafounders are already partially inbred and related to each other [12]. The metafounder-layer is followed by a first layer consisting of real animals: the pedigree base (Layer 3). Because animals of the pedigree base are in most cases not identical with strata-founders, they cannot be considered as part of the metafounders-layer. The fourth layer consists of genotyped animals. This layer provides the information required to estimate the characteristics at the metafounder-layer (i.e., allele frequencies). To be able to consistently transfer this information across all layers, the genotyped animals need to be connected to the pedigree base (link 3) and there should be some degree of knowledge of the pedigree base’s relationship to the metafounders (link 2).

Applying the metafounders concept in a population with a long history of crossbreeding and admixture like the European Brown Swiss population lead to some obstacles. The information needed to define metafounders was only available from the genotyped animals. When assuming two metafounders for Brown Swiss (European and US origin), pedigrees would need to be traced back to the 1960s, before admixture started. Most pedigrees of the current Brown Swiss population could not be traced back to that period, thus no information regarding the admixture level of the pedigree base was available. Without this information, an assignment of the pedigree base to the metafounders remained arbitrary. Because of that missing link, the information regarding metafounder allele frequencies could not be passed on from the genotyped animals via the pedigree base to the metafounder (links 3 and 2 fail).

In current literature, metafounders are defined similar to unknown parent groups (UPG) [12–15, 17, 18, 55], by assigning pedigree-founders to metafounders based on information on birth-years and/or known or assumed origin. This approach showed in previous studies varying results on bias. When modelling the same number of metafounders and UPG in routine evaluation, Kudinov et al. [17] found only slight improvements in bias. Macedo et al. [14] found that only a combination of metafounders and a systematic data cut gave unbiased results in ssGBLUP. Other authors reported bias due to unbalanced definition of metafounders [56] or UPG [55]. Fikse [57] addressed considerations in establishing UPG and problems with assigning UPG based on incorrect information. Using data from Lacaune sheep, Macedo et al. [14] reported ~ 8% missing pedigrees. In dairy cattle especially dam information is frequently missing [58]. In a situation where genomic information of animals in a population is readily available, defining metafounders based on (incomplete) pedigree information alone seems to be insufficient and might lead to a situation where the existing stratification of the population is not sufficiently detected. In a worst-case scenario, the information represented by one true metafounder might be divided into several UPG/metafounder and be greatly diluted or eventually be lost. In our case, animals registered as OBV were not part of the routine breeding value estimation. If OBV animals would be included in the genomic evaluation, based on their ISO-Codes, they would be assigned to an UPG comprising unknown parents from Switzerland. This would not reflect the true stratification that requires a separate group for OBV. When estimating allele frequencies for those Swiss groups, the resulting estimates would most likely miss the relevant aspects of the OBV group, working instead with a mean of modern Brown Swiss and OBV animals. This situation would be comparable to the situation found in the analysis of S1, where $k$ = 2 did not identify Brown Swiss and OBV, but a stratification within the Brown Swiss cluster, clustering OBV together with US-influenced animals. This yielded no improvements compared to the initial situation, only the detection of OBV as a distinct stratum improved the compatibility of ${\mathbf{A}}^{{\varvec{\Gamma}}}$ and $\mathbf{G}$ considerably (Table 4).

In this context, it is interesting that PCA and ADMIXTURE both identified individuals in the data as OBV that were registered as members of the modern Brown Swiss. Again, this information would have been lost in the usual metafounder approach. This might at least partly explain why models using metafounders showed no conclusive improvements in ssGBLUP compared to approaches with UPG [12–15, 17, 18, 55].

### Estimation of ${\varvec{\Gamma}}$

We were not able to find any reference using information from population structure analysis to define metafounders and estimate characteristic allele frequencies. Legarra et al. [12] proposed two methods to estimate ${\varvec{\Gamma}}$: a maximum likelihood method and a method of moments based on summary statistics. Garcia-Baccino et al. [43] presented a GLS method to yield unbiased results. We used a $\mathbf{Q}$ matrix estimated from an analysis of population structure in combination with an approach to estimate base allele frequencies proposed by Gengler et al. [44] and adapted as proposed by Aldridge et al. [45]. To establish a homogenous base population, only animals with at least 85% of their ancestry traceable to a base set to 1985 were used for the estimation of allele frequencies. When *k* increased, we observed an increasing number of estimates outside the parameter space. This was consistent with population genetic theory, as far as in divergent lines alleles become fixed [2]. However, this could also be a consequence of reducing effective sample sizes for the estimation of metafounder frequencies. Nevertheless, since the elements of ${\varvec{\Gamma}}$ are functions of summary statistics across many markers (e.g., average expected heterozygosity across 42k markers) we assume that decreasing sample sizes did not have a strong impact on the results.

### Transfer of Information

We presented an approximate approach to construct ${\mathbf{A}}^{{\varvec{\Gamma}}}$, with separate steps for the introduction of stratification information into ${\mathbf{A}}_{g}$ and the rescaling of the relationship matrix to a different genetic base. This way, we detangled two fundamental aspects that were somewhat obscure in Legarra et al.’s [12] original description of the approach. When comparing our approach to the corresponding submatrix derived by the approximation given in Legarra et al. [12] we found our approximation to be more comprehensible, and slightly more precise with respect to inbreeding coefficients but otherwise providing similar results (see Additional file 3). In contrast to the conclusions of Legarra et al. [12], we conclude that a consistent approximation of ${\mathbf{A}}^{{\varvec{\Gamma}}}$ using a general expression of the form ${\mathbf{A}}^{{\varvec{\Gamma}}}\approx {\mathbf{A}}_{g}+ \mathbf{Q}{\varvec{\Gamma}}\mathbf{Q}^{\prime}$ is reasonable, when aspects of rescaling are correctly taken into account, as we showed in the "Methods". A model, where strata-effects are random effects with covariance-matrix ${\varvec{\Gamma}}$ like for example proposed in [36], would be an appropriate choice in such a situation. Moreover, deriving information for ${\varvec{\Gamma}}$ from genotypes and adding this information to ${\mathbf{A}}_{g}$ to improve the compatibility to **G** would in reverse suggest that matrix **G** in the presence of stratification is approximately of the form ${\mathbf{G}}_{\mathbf{A}}+ \mathbf{Q}{\varvec{\Gamma}}\mathbf{Q}^{{\prime}}$, where ${\mathbf{G}}_{\mathbf{A}}$ is a genomic relationship matrix free of stratum information [36].

### Shortcomings

An evaluation of the performance of our optimum ${\mathbf{A}}^{{\varvec{\Gamma}}}$ in routine genetic evaluation was beyond the scope of this paper. Whether the obtained ${\mathbf{A}}^{{\varvec{\Gamma}}}$ improves ssGBLUP will be subject of further research. Since we estimated $\mathbf{Q}$ only for genotyped animals, the implementation of our approach in ssGBLUP is not straightforward. To achieve an implementation, different sources of information must be connected: animals with phenotypes only, animals with genotypes and phenotypes, and animals in their pedigree. Assigning animals at the end of a pedigree to UPG/metafounders (and tracing contributions through the pedigree) using traditional strategies is not directly applicable to our approach. In order to achieve that, matrix $\mathbf{Q}$ would have to be extrapolated to non-genotyped animals in the pedigree. At present we do not see a feasible way to extrapolate $\mathbf{Q}$ to non-genotyped animals beyond a quite general projection based on pedigree information. Another aspect is in line with concepts developed by Plieschke et al. [36]. In their study, instead of adding information to $\mathbf{A}$, $\mathbf{G}$ was manipulated to match ${\mathbf{A}}_{g}$ by subtracting stratification information. However, this stratification information might better be modeled independently. Both of these important aspects were beyond the scope of the current investigation and will hopefully be addressed in future investigations.

## Conclusion

The goal of this study was to improve the compatibility of matrices ${\mathbf{A}}_{g}$ and $\mathbf{G}$ by using stratification information directly derived from genotype data. We used ADMIXTURE to identify strata and we used methodology described in the metafounder concept to introduce this information into ${\mathbf{A}}_{g}$. Evaluation of this process was based on the realized improvements obtained by visual inspection of graphs from PCA, by regression analysis and through the comparison of the mean and mean diagonal values of both matrices.

Adding stratification information to ${\mathbf{A}}_{g}$ improved the compatibility of resulting ${\mathbf{A}}^{{\varvec{\Gamma}}}$ and $\mathbf{G}$ considerably compared to the initial situation. An interpretation of the optimum $k$ as identified by the evaluation criteria ($k$ = 24 for S2 and S3) was not straightforward and was not the aim of this study. For our purpose, we considered each stratification to be relevant as long as it improved the compatibility of ${\mathbf{A}}_{g}$ and $\mathbf{G}$. ADMIXTURE can be a helpful tool to identify such stratification in the data, but restriction of data in analysis is necessary when applying ADMIXTURE in modern dairy populations which are made up of large half-sib groups.

An alternative approach to approximate ${\mathbf{A}}^{{\varvec{\Gamma}}}$ was successfully applied and gave results that were consistent with the original approach of Legarra et al. [12], finding a clear optimum for the compatibility of both matrices. Moreover, this study gives a clear and comprehensible explanation of the theoretical background of metafounders, illustrated by analysis of stratification present in the European Brown Swiss population. Future work will focus on the implementation of this unsupervised approach of defining metafounders into routine evaluation.

## Supplementary Information

**Additional file 1: **Figure S1: PCA for PC3 and PC4 of large half-sib families corresponding to stratification identified by ADMIXTURE in the full set of genotypes (S1). Separable groups within modern Brown Swiss relate to specific sires. Shape indicates parentage of a specific sire. Larger Shapes indicate the specific sires themselves. Registered OBV are all one shape. Color is according to assigned stratification to a specific animal.

**Additional file 2. **

**Additional file 3. **Comparison of Legarra approximation and new approximation of ${\text{A}}^{\Gamma }$.

## Data Availability

Genotypes are property of the breeding organisations / Intergenomics and therefore not publicly available.
