# Supplementary figures and images for: Definition of metafounders based on population structure analysis
Source: Genet Sel Evol. 2024 Jun 6;56:43. doi: 10.1186/s12711-024-00913-7 (PMC11536677; doi:10.1186/s12711-024-00913-7)

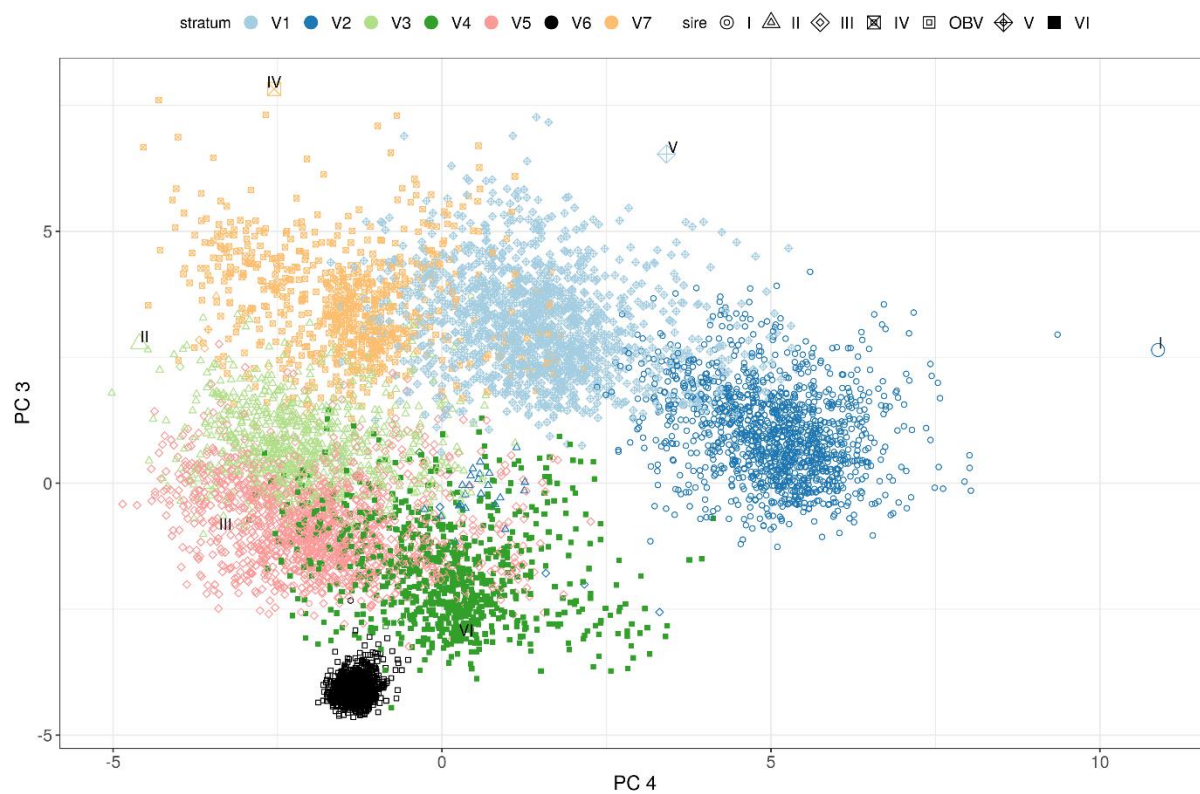

Supplement: Supplementary file 1 — Additional file 1: Figure S1: PCA for PC3 and PC4 of large half-sib families corresponding to stratification identified by ADMIXTURE in the full set of genotypes (S1). Separable groups within modern Brown Swiss relate to specific sires. Shape indicates parentage of a specific sire. Larger Shapes indicate the specific sires themselves. Registered OBV are all one shape. Color is according to assigned stratification to a specific animal. [file 12711_2024_913_MOESM1_ESM.pdf]
