# Supplementary material for: Definition of metafounders based on population structure analysis
Source: Genet Sel Evol. 2024 Jun 6;56:43. doi: 10.1186/s12711-024-00913-7 (PMC11536677; doi:10.1186/s12711-024-00913-7)
Supplement: Supplementary file 2 — Additional file 2. [file 12711_2024_913_MOESM2_ESM.pdf]

Additional File 2

Table S1: Detailed structure of the full data set (S1).

Birth year	1950-1959		1960-1969		1970-1979		1980-1989		1990-1999		2000-2009		2010-2019		2020-2022	
	male	female	male	female	male	female	male	female	male	female	male	female	male	female	male	female
CHE	0	0	0	0	95	0	186	0	624	0	1024	3	2395	14	605	1
DEA	0	0	1	0	21	1	80	0	905	6	1364	1037	15,300	35,384	4495	14,077
ITA	0	0	2	0	59	0	161	0	513	1	522	0	1239	3	34	1
OBV	0	0	23	0	38	0	14	0	118	0	199	0	561	2	224	1
OTHER	0	0	0	0	1	0	9	0	143	0	203	8	1071	31	294	10
USACAN	3	0	22	0	57	0	123	0	344	0	448	0	759	15	375	0

Assignment was done by country of origin: CHE = Switzerland, DEA = Germany and Austria, ITA = Italy, OBV = Animals recorded as Original Braunvieh, USACAN = United States of America and Canada, OTHER = Other country of origin.

Table S2: Results for S3 (additional sample with reduced relationship).

k	a	b	R^2	$\overline{G} - \overline{A^{\Gamma}}$	$\overline{diag(G)} - \overline{diag(A^{\Gamma})}$
2	-0.026	1.059	0.802	-0.019	-0.024
3	-0.020	1.071	0.784	-0.034	-0.036
4	-0.051	1.109	0.793	-0.033	-0.039
5	-0.059	1.117	0.794	-0.031	-0.038
6	-0.053	1.104	0.797	-0.026	-0.036
7	-0.054	1.104	0.797	-0.026	-0.037
8	-0.061	1.110	0.800	-0.024	-0.037
9	-0.056	1.099	0.800	-0.019	-0.034
10	-0.061	1.098	0.803	-0.014	-0.031
15	-0.071	1.094	0.811	-0.001	-0.021
20	-0.054	1.056	0.814	0.012	-0.010
21	-0.050	1.047	0.817	0.014	-0.009
22	-0.047	1.041	0.817	0.016	-0.007
23	-0.032	1.017	0.818	0.019	-0.004
24	-0.022	0.999	0.817	0.023	0.000
25	-0.022	0.996	0.817	0.025	0.001
30	-0.021	0.980	0.811	0.036	0.011
35	-0.014	0.956	0.805	0.047	0.021
40	-0.050	0.997	0.809	0.052	0.028

k = number of stratifications considered, a = intercept, b = slope of the regression, R^2 = fit of the regression, G = genomic relationship matrix, A^{Γ} = numerator relationship matrix amended by stratification information, $\overline{G} - \overline{A^{\Gamma}}$ = Difference of means of both matrices, $\overline{diag(G)} - \overline{diag(A^{\Gamma})}$ = Difference of the means of the diagonals of both matrices.

Table S3: Results for S4 (additional sample of 4152 randomly selected animals).

**k**	**a**	**b**	**R^2**	$\overline{G} - \overline{A^{\Gamma}}$	$\overline{diag(G)} - \overline{diag(A^{\Gamma})}$
2	0.339	0.531	0.639	0.019	0.015
3	0.258	0.652	0.698	0.009	0.004
4	0.226	0.696	0.700	0.007	0.000
5	0.213	0.712	0.720	0.008	0.000
6	0.206	0.717	0.721	0.010	0.000
7	0.202	0.722	0.721	0.011	0.000
8	-0.003	1.033	0.809	-0.022	-0.033
9	-0.006	1.032	0.810	-0.018	-0.032
10	-0.046	1.083	0.814	-0.018	-0.034
15	-0.059	1.082	0.814	-0.004	-0.027
20	-0.075	1.083	0.816	0.011	-0.017
21	-0.075	1.082	0.814	0.012	-0.016
22	-0.075	1.076	0.817	0.017	-0.012
23	-0.078	1.079	0.815	0.017	-0.012
24	-0.073	1.070	0.814	0.020	-0.010
25	-0.074	1.069	0.814	0.022	-0.008
30	-0.084	1.069	0.815	0.031	-0.001
35	-0.092	1.069	0.813	0.039	0.007
40	-0.096	1.065	0.810	0.047	0.014

k = number of stratifications considered, a = intercept, b = slope of the regression, R^2 = fit of the regression, G = genomic relationship matrix, A^{Γ} = numerator relationship matrix amended by stratification information, $\overline{G} - \overline{A^{\Gamma}}$ = Difference of means of both matrices, $\overline{diag(G)} - \overline{diag(A^{\Gamma})}$ = Difference of the means of the diagonals of both matrices.
