# Supplementary material for: Definition of metafounders based on population structure analysis
Source: Genet Sel Evol. 2024 Jun 6;56:43. doi: 10.1186/s12711-024-00913-7 (PMC11536677; doi:10.1186/s12711-024-00913-7)
Supplement: Supplementary file 3 — Additional file 3. Comparison of Legarra approximation and new approximation of ${\text{A}}^{\Gamma }$AΓ. [file 12711_2024_913_MOESM3_ESM.pdf]

A comparison of the approximation proposed by Legarra et al. [12] and the approximation presented in this paper:

The comparison was based on a small example pedigree taken from Legarra et al. [12] to illustrate the different aspects of both approaches.

Example Pedigree:

animal	sire	dam
MF1	NA	NA
MF2	NA	NA
F1	MF1	MF1
F2	MF1	MF1
F3	MF1	MF2
F4	MF2	MF2
F5	MF2	MF2
A1	F1	F24
A2	F2	F3
A3	F4	F5
A4	A1	A2
A5	F3	A3
A6	A1	F3
A7	A4	A5

MF1 and MF2 are metafounders that were included in the pedigree. Animals F1 to F5 are the pedigree base, where animal F3 is a mixture of the two MF. The following $\mathbf{\Gamma}$ matrix is assumed:

$$\begin{bmatrix} 0.10 & 0.05 \\ 0.05 & 0.20 \end{bmatrix}$$

$\mathbf{A}^{\mathbf{\Gamma}}$ following the ‚tabular method‘ including the two MF:

	MF1	MF2	F1	F2	F3	F4	F5	F6	F7	A1	A2	A3	A4	A5
MF1	0.10	0.05	0.10	0.10	0.08	0.05	0.05	0.10	0.09	0.05	0.09	0.06	0.09	0.08
MF2	0.05	0.20	0.05	0.05	0.12	0.20	0.20	0.05	0.09	0.20	0.07	0.16	0.09	0.12
F1	0.10	0.05	1.05	0.10	0.08	0.05	0.05	0.58	0.09	0.05	0.33	0.06	0.33	0.20
F2	0.10	0.05	0.10	1.05	0.08	0.05	0.05	0.58	0.56	0.05	0.57	0.06	0.33	0.32
F3	0.08	0.12	0.08	0.08	1.02	0.12	0.12	0.08	0.55	0.12	0.31	0.57	0.55	0.44
F4	0.05	0.20	0.05	0.05	0.12	1.10	0.20	0.05	0.09	0.65	0.07	0.39	0.09	0.23
F5	0.05	0.20	0.05	0.05	0.12	0.20	1.10	0.05	0.09	0.65	0.07	0.39	0.09	0.23
F6	0.10	0.05	0.58	0.58	0.08	0.05	0.05	1.05	0.33	0.05	0.69	0.06	0.56	0.38
F7	0.09	0.09	0.09	0.56	0.55	0.09	0.09	0.33	1.04	0.09	0.68	0.32	0.44	0.50
A1	0.05	0.20	0.05	0.05	0.12	0.65	0.65	0.05	0.09	1.10	0.07	0.61	0.09	0.34
A2	0.09	0.07	0.33	0.57	0.31	0.07	0.07	0.69	0.68	0.07	1.16	0.19	0.50	0.68
A3	0.06	0.16	0.06	0.06	0.57	0.39	0.39	0.06	0.32	0.61	0.19	1.06	0.32	0.63
A4	0.09	0.09	0.33	0.33	0.55	0.09	0.09	0.56	0.44	0.09	0.50	0.32	1.04	0.41
A5	0.08	0.12	0.20	0.32	0.44	0.23	0.23	0.38	0.50	0.34	0.68	0.63	0.41	1.10

For the calculation of the approximation, the **Q**-matrix of animals in the pedigree is needed. Tracing contributions of the two MF through the pedigree, **Q** is:

	MF1	MF2
F1	1.0000	0.0000
F2	1.0000	0.0000
F3	0.5000	0.5000
F4	0.0000	1.0000
F5	0.0000	1.0000
F6	1.0000	0.0000
F7	0.7500	0.2500
A1	0.0000	1.0000
A2	0.8750	0.1250
A3	0.2500	0.7500
A4	0.7500	0.2500
A5	0.5625	0.4375

Legarra approximation

With this $\mathbf{Q}$ an approximation of $\mathbf{A}^{\boldsymbol{\Gamma}}$ as proposed by Legarra et al.[12]:

$$\mathbf{A}^{\boldsymbol{\Gamma}} \approx \mathbf{A}(\mathbf{I} - \mathbf{0.5} * \boldsymbol{diag}(\mathbf{Q}\boldsymbol{\Gamma}\mathbf{Q}') + \mathbf{Q}\boldsymbol{\Gamma}\mathbf{Q}'$$

was calculated (in the following comparisons the exact version is presented first, followed by results of the approximation. Results are only for animals of the pedigree).

Comparisons for animals in the pedigree:

Exact $\mathbf{A}^{\boldsymbol{\Gamma}}$

	F1	F2	F3	F4	F5	F6	F7	A1	A2	A3	A4	A5
F1	1.050	0.100	0.075	0.050	0.050	0.575	0.088	0.050	0.331	0.062	0.325	0.197
F2	0.100	1.050	0.075	0.050	0.050	0.575	0.562	0.050	0.569	0.062	0.325	0.316
F3	0.075	0.075	1.025	0.125	0.125	0.075	0.550	0.125	0.312	0.575	0.550	0.444
F4	0.050	0.050	0.125	1.100	0.200	0.050	0.088	0.650	0.069	0.388	0.088	0.228
F5	0.050	0.050	0.125	0.200	1.100	0.050	0.088	0.650	0.069	0.388	0.088	0.228
F6	0.575	0.575	0.075	0.050	0.050	1.050	0.325	0.050	0.688	0.062	0.562	0.375
F7	0.088	0.562	0.550	0.088	0.088	0.325	1.038	0.088	0.681	0.319	0.438	0.500
A1	0.050	0.050	0.125	0.650	0.650	0.050	0.088	1.100	0.069	0.612	0.088	0.341
A2	0.331	0.569	0.312	0.069	0.069	0.688	0.681	0.069	1.163	0.191	0.500	0.677
A3	0.062	0.062	0.575	0.388	0.388	0.062	0.319	0.612	0.191	1.062	0.319	0.627
A4	0.325	0.325	0.550	0.088	0.088	0.562	0.438	0.088	0.500	0.319	1.038	0.409
A5	0.197	0.316	0.444	0.228	0.228	0.375	0.500	0.341	0.677	0.627	0.409	1.095

Legarra Approximation of $\mathbf{A}^{\boldsymbol{\Gamma}}$

	F1	F2	F3	F4	F5	F6	F7	A1	A2	A3	A4	A5
F1	1.050	0.100	0.075	0.050	0.050	0.575	0.088	0.050	0.332	0.062	0.327	0.197
F2	0.100	1.050	0.075	0.050	0.050	0.575	0.566	0.050	0.571	0.062	0.327	0.316
F3	0.075	0.075	1.050	0.125	0.125	0.075	0.566	0.125	0.320	0.578	0.566	0.454
F4	0.050	0.050	0.125	1.100	0.200	0.050	0.088	0.650	0.069	0.395	0.088	0.235
F5	0.050	0.050	0.125	0.200	1.100	0.050	0.088	0.650	0.069	0.395	0.088	0.235
F6	0.575	0.575	0.075	0.050	0.050	1.050	0.327	0.050	0.690	0.062	0.566	0.376
F7	0.088	0.562	0.562	0.088	0.088	0.325	1.044	0.088	0.684	0.320	0.446	0.504
A1	0.050	0.050	0.125	0.650	0.650	0.050	0.088	1.100	0.069	0.628	0.088	0.354
A2	0.331	0.569	0.319	0.069	0.069	0.688	0.685	0.069	1.165	0.191	0.506	0.678
A3	0.062	0.062	0.588	0.388	0.388	0.062	0.327	0.612	0.194	1.069	0.327	0.642
A4	0.325	0.325	0.562	0.088	0.088	0.562	0.446	0.088	0.505	0.320	1.044	0.415
A5	0.197	0.316	0.453	0.228	0.228	0.375	0.506	0.341	0.679	0.630	0.416	1.107

Correlation of all elements of the matrices is > 0.999.
Comparison of diagonal elements of both matrices:

	F1	F2	F3	F4	F5	F6	F7	A1	A2	A3	A4	A5
Exact:	1.05	1.05	1.025	1.1	1.1	1.05	1.03750	1.1	1.162500	1.06250	1.03750	1.095312
Legarra:	1.05	1.05	1.050	1.1	1.1	1.05	1.04375	1.1	1.164648	1.06875	1.04375	1.106812

Especially for animal F3 (which has contributions of 0.5 of each MF) a relatively large difference (0.025) in the diagonal element can be observed. The Legarra approximation overestimates the inbreeding coefficient of the admixed animal.

New approximation

For the approach presented in the paper $\boldsymbol{\Gamma}$ is rescaled to the heterozygosity corresponding to a pivotal γ ($\boldsymbol{\Gamma}$ and $\mathbf{A}$ pointing to the same base/heterozygosity). $\boldsymbol{\Gamma}$ is transformed into a relationship matrix and $\mathbf{A}^{\boldsymbol{\Gamma}}$ is calculated and finally rescaled to a base of maximum heterozygosity (0.5) to be on the same scale as $\mathbf{G}$.

Assuming a pivotal γ of 0.1, the corresponding heterozygosity ($H_B = 0.5 - 0.25\gamma$) is 0.475. Rescaling $\boldsymbol{\Gamma}$ to a base of 0.475 yields:

$$\begin{bmatrix} 0.000 & -0.053 \\ -0.053 & 0.105 \end{bmatrix}$$

$\boldsymbol{\Gamma}$ in the form of a relationship matrix (diagonal is 1 + 0.5*self-relationship):

$$\begin{bmatrix} 1.000 & -0.053 \\ -0.053 & 1.053 \end{bmatrix}$$

Now $\mathbf{A}^{\boldsymbol{\Gamma}} \approx \mathbf{A} + \mathbf{Q}(\boldsymbol{\Gamma} - \mathbf{diag}(\mathbf{2}))\mathbf{Q}'$ can be calculated:

	F1	F2	F3	F4	F5	F6	F7	A1	A2	A3	A4	A5
F1	1.000	0.000	-0.026	-0.053	-0.053	0.500	-0.013	-0.053	0.243	-0.039	0.237	0.102
F2	0.000	1.000	-0.026	-0.053	-0.053	0.500	0.487	-0.053	0.493	-0.039	0.237	0.227
F3	-0.026	-0.026	0.987	0.000	0.000	-0.026	0.480	0.000	0.227	0.493	0.480	0.360
F4	-0.053	-0.053	0.000	1.053	0.053	-0.053	-0.026	0.553	-0.039	0.276	-0.026	0.118
F5	-0.053	-0.053	0.000	0.053	1.053	-0.053	-0.026	0.553	-0.039	0.276	-0.026	0.118
F6	0.500	0.500	-0.026	-0.053	-0.053	1.000	0.237	-0.053	0.618	-0.039	0.487	0.289
F7	-0.013	0.487	0.480	-0.026	-0.026	0.237	0.984	-0.026	0.610	0.227	0.359	0.419
A1	-0.053	-0.053	0.000	0.553	0.553	-0.053	-0.026	1.053	-0.039	0.526	-0.026	0.243
A2	0.243	0.493	0.227	-0.039	-0.039	0.618	0.610	-0.039	1.114	0.094	0.423	0.604
A3	-0.039	-0.039	0.493	0.276	0.276	-0.039	0.227	0.526	0.094	1.010	0.227	0.552
A4	0.237	0.237	0.480	-0.026	-0.026	0.487	0.359	-0.026	0.423	0.227	0.984	0.325
A5	0.102	0.227	0.360	0.118	0.118	0.289	0.419	0.243	0.604	0.552	0.325	1.047

And rescaled to a base of 0.5:

	F1	F2	F3	F4	F5	F6	F7	A1	A2	A3	A4	A5
F1	1.050	0.100	0.075	0.050	0.050	0.575	0.088	0.050	0.331	0.063	0.325	0.197
F2	0.100	1.050	0.075	0.050	0.050	0.575	0.563	0.050	0.569	0.063	0.325	0.316
F3	0.075	0.075	1.038	0.100	0.100	0.075	0.556	0.100	0.316	0.569	0.556	0.442
F4	0.050	0.050	0.100	1.100	0.150	0.050	0.075	0.625	0.063	0.363	0.075	0.213
F5	0.050	0.050	0.100	0.150	1.100	0.050	0.075	0.625	0.063	0.363	0.075	0.213
F6	0.575	0.575	0.075	0.050	0.050	1.050	0.325	0.050	0.688	0.063	0.563	0.375
F7	0.088	0.563	0.556	0.075	0.075	0.325	1.034	0.075	0.680	0.316	0.441	0.498
A1	0.050	0.050	0.100	0.625	0.625	0.050	0.075	1.100	0.063	0.600	0.075	0.331
A2	0.331	0.569	0.316	0.063	0.063	0.688	0.680	0.063	1.159	0.189	0.502	0.674
A3	0.063	0.063	0.569	0.363	0.363	0.063	0.316	0.600	0.189	1.059	0.316	0.624
A4	0.325	0.325	0.556	0.075	0.075	0.563	0.441	0.075	0.502	0.316	1.034	0.409
A5	0.197	0.316	0.442	0.213	0.213	0.375	0.498	0.331	0.674	0.624	0.409	1.094

Correlation of all elements of this approximation to the exact $\mathbf{A}^{\boldsymbol{\Gamma}}$ is > 0.999.

Comparing the diagonal elements of all three matrices (exact on the top, Legarra approximation in the middle, new approximation at the bottom), the new approximation shows overall a slightly better compliance to the exact version especially with admixed animals.

	F1	F2	F3	F4	F5	F6	F7	A1	A2	A3	A4	A5
Exact:	1.05	1.05	1.025	1.1	1.1	1.05	1.038	1.1	1.163	1.062	1.038	1.095
Legarra:	1.05	1.05	1.050	1.1	1.1	1.05	1.044	1.1	1.165	1.069	1.044	1.107
New:	1.05	1.05	1.038	1.1	1.1	1.05	1.034	1.1	1.159	1.059	1.034	1.094

An approximation of $\mathbf{A}^{\boldsymbol{\Gamma}}$ as described seems to be reasonably accurate. The example illustrates that rescaling is conceptually separable from the introduction of stratification information.
